# Current status of phylogenetic studies on ciliated protists (Alveolata, Protozoa, Ciliophora) by the OUC-group: advances, challenges and future perspectives

**DOI:** 10.1007/s42995-025-00326-5

**Published:** 2025-11-21

**Authors:** Feng Gao, Yang Bai, Yong Chi, Xiaochen Feng, Chunyu Lian, Borong Lu, Xiaotian Luo, Mingzhen Ma, Congcong Wang, Yurui Wang, Zhe Wang, Gongaote Zhang, Saleh A. Al-Farraj, Alan Warren, Weibo Song

**Affiliations:** 1 Key Laboratory of Evolution and Marine Biodiversity (Ministry of Education), and Institute of Evolution and Marine Biodiversity, Ocean University of China, Qingdao, 266003 China; 2 Laboratory for Marine Biology and Biotechnology, Qingdao Marine Science and Technology Center, Qingdao, 266237 China; 3 Ministry of Education Key Laboratory of Ecology and Resource Use of the Mongolia Plateau, Inner Mongolia University, Hohhot, 010021 China; 4 Marine College, Shandong University, Weihai, 264209 China; 5 College of Life Science and Technology, Harbin Normal University, Harbin, 150025 China; 6 State Key Laboratory of Lake and Watershed Science for Water Security, Institute of Hydrobiology, Chinese Academy of Sciences, Wuhan, 430072 China; 7 Ningbo Yonghuanyuan Environmental Engineering and Technology Co., Ltd, Ningbo, 315012 China; 8 Laboratory of Biodiversity and Evolution of Protozoa in Wetland, College of Life Sciences, Shaanxi Normal University, Xi’an, 710119 China; 9 Department of Zoology, King Saud University, 11451 Riyadh, Saudi Arabia; 10 Department of Life Sciences, Natural History Museum, London, SW7 5BD UK

**Keywords:** Ciliates, On-going challenges, Phylogeny and evolution, Phylogenomics, rDNA

## Abstract

Ciliated protists (ciliates) represent a morphologically and genetically diverse group of single-celled eukaryotes, the phylogeny of which is critical for understanding eukaryotic evolution. Through international collaborations, the Laboratory of Protozoology at Ocean University of China (OUC-group) has conducted detailed research on ciliate phylogeny based on expanded taxonomic sampling, employing single gene as well as multi-gene markers, and phylogenomic datasets. We have systematically investigated > 1000 ciliate species spanning ~ 40 orders, sampled from diverse biotopes including marine environments in China seas and freshwater wetlands. This comprehensive sampling has generated three key datasets: (1) genomic DNA extracts from ~ 2600 strains, (2) ~ 2300 sequences of marker genes, and (3) single-cell genomic and/or transcriptomic datasets from ~ 120 species. Based on these datasets, the phylogenetic relationships covering all classes and most orders have been thoroughly reconstructed and investigated, resulting in the establishment of 93 new supraspecies taxa comprising two classes (Mesodiniea and Protocruziea), two subclasses (Protohypotrichia and Synhymenia), two orders (Wilbertomorphida and Lynnellida), 11 families, and 76 genera. Moreover, we have reconstructed a genome-scale tree of life for ciliates and provided an updated classification of the phylum Ciliophora. Furthermore, based on the robust phylogenetic tree of ciliates, we provide more reliable estimates for the origins and divergence times of the main ciliate groups. Future studies integrating advanced genomics, innovations in culturing and interdisciplinary applications will refine the ciliate tree of life, with broader impacts for our understanding of eukaryotic evolution and biodiversity.

## Introduction

Ciliates (phylum Ciliophora), which emerged approximately one billion years ago, are an ecologically important group of single-celled eukaryotes that play crucial roles in microbial food webs (Lynn 2008; Parfrey et al. 2011). They have undergone extensive diversification resulting in a high diversity of species that are adapted to a wide range of ecological niches (Song et al. 2003, 2009). In addition, ciliates show many intriguing and unique biological features, e.g., possessing both a germline micronucleus and a somatic macronucleus within the same cell, and performing a special sexual process known as conjugation, during which genome-wide DNA rearrangement occurs, resulting in fragmented macronuclear genomes (reviewed in Gao et al. 2023). Hence, some ciliates (e.g., *Tetrahymena, Paramecium*) have been used as important model organisms in a wide range of biological studies, resulting in many significant discoveries, including self-splicing RNAs, telomerase, and epigenetic modifications (Greider and Blackburn 1985; Kruger et al. 1982; Wang et al. 2017b).

A robust and accurate phylogeny of ciliates is essential for elucidating the evolutionary origins and diversification of their biological features and mechanisms. Such phylogenies also have a significant impact on reconstructions of the broader tree of life, as missing or inadequately sampled lineages can lead to topological instability and compromise phylogenetic inference (Heath et al. 2008). Historically, systematic and phylogenetic studies of ciliates have relied primarily on morphological, morphogenetic and ultrastructural data, with the most widely accepted classification system of the phylum Ciliophora comprising three classes (Kinetofragminophora, Oligohymenophora, and Polyhymenophora), 27 orders and 207 families (Corliss 1979). However, the presence of convergent evolution (the independent emergence of similar structures in unrelated lineages) has often led to misclassifications. Since the 1980s, the development of polymerase chain reaction (PCR) and Sanger sequencing technologies established the small subunit ribosomal RNA gene (SSU rDNA) as a powerful molecular marker for resolving eukaryotic phylogenies. Following the initial characterization of the SSU rDNA in two hypotrichous ciliates (Elwood et al. 1985), phylogenetic analyses based on this molecular marker have greatly advanced our understanding of ciliate evolution (e.g., Gao et al. 2016, 2017). However, evolutionary relationships within ciliates remain contentious for two reasons: (1) the SSU rDNA molecular marker provides insufficient resolution of the deep branches and closely related species or rapidly evolving groups; (2) molecular data are limited to a relatively small number of organisms, with underrepresented lineages particularly lacking. Recent investigations using multi-gene markers and phylogenomic datasets have significantly improved our understanding of ciliate phylogeny, resolving long-standing uncertainties regarding their evolutionary relationships (Gentekaki et al. 2014; Wang et al. 2021a). Currently, the phylum Ciliophora comprises 17 classes, 73 orders and about 337 families (Wang et al. In press).

Since the 1990s, the research group at the Laboratory of Protozoology at the Ocean University of China (OUC-group) has conducted extensive phylogenetic studies of ciliates in collaborations with researchers worldwide, supported by multiple national and international research programs. Our investigations have systematically characterized over 1000 ciliate species representing approximately 40 orders, which were sampled from various biotopes in China, including freshwater wetlands and marine environments in coastal regions (Song et al. 2003, 2009). This sampling strategy has produced three key datasets: (1) genomic DNA extracts from about 2600 strains; (2) over 2300 phylogenetically informative sequences from marker genes (SSU rDNA, LSU rDNA, ITS-5.8S, etc.); and (3) single-cell genomic and/or transcriptomic data from about 120 representative species (e.g., Gao et al. 2016, 2017; Lyu et al. 2024). Based on these datasets, the phylogenetic relationships covering all classes and most orders have been analyzed in detail. The present paper summarizes the main findings of the OUC-group’s studies on major ciliate lineages.

### Phylogenetic studies of the class Karyorelictea (Fig. 1)

The class Karyorelictea Corliss, 1974 represents a distinct phylogenetic lineage that is often considered “primitive” due to its ancestral morphological and nuclear characteristics, i.e., (1) the absence of cirri, (2) the reduced oral ciliature, and (3) the unique inability of the somatic nuclei to divide (Lynn 2008; Mazei et al. 2009; Yan et al. 2017, 2019). Since the early part of this century, phylogenetic relationships within this class have been investigated using integrative data, particularly with the availability of nuclear gene markers such as SSU rDNA (Ma et al. 2021a, b, 2025; Mazei et al. 2009; Wang et al. 2019b; Xu et al. 2011b, 2012, 2014; Yan et al. 2013, 2015, 2016).

**Fig. 1:**
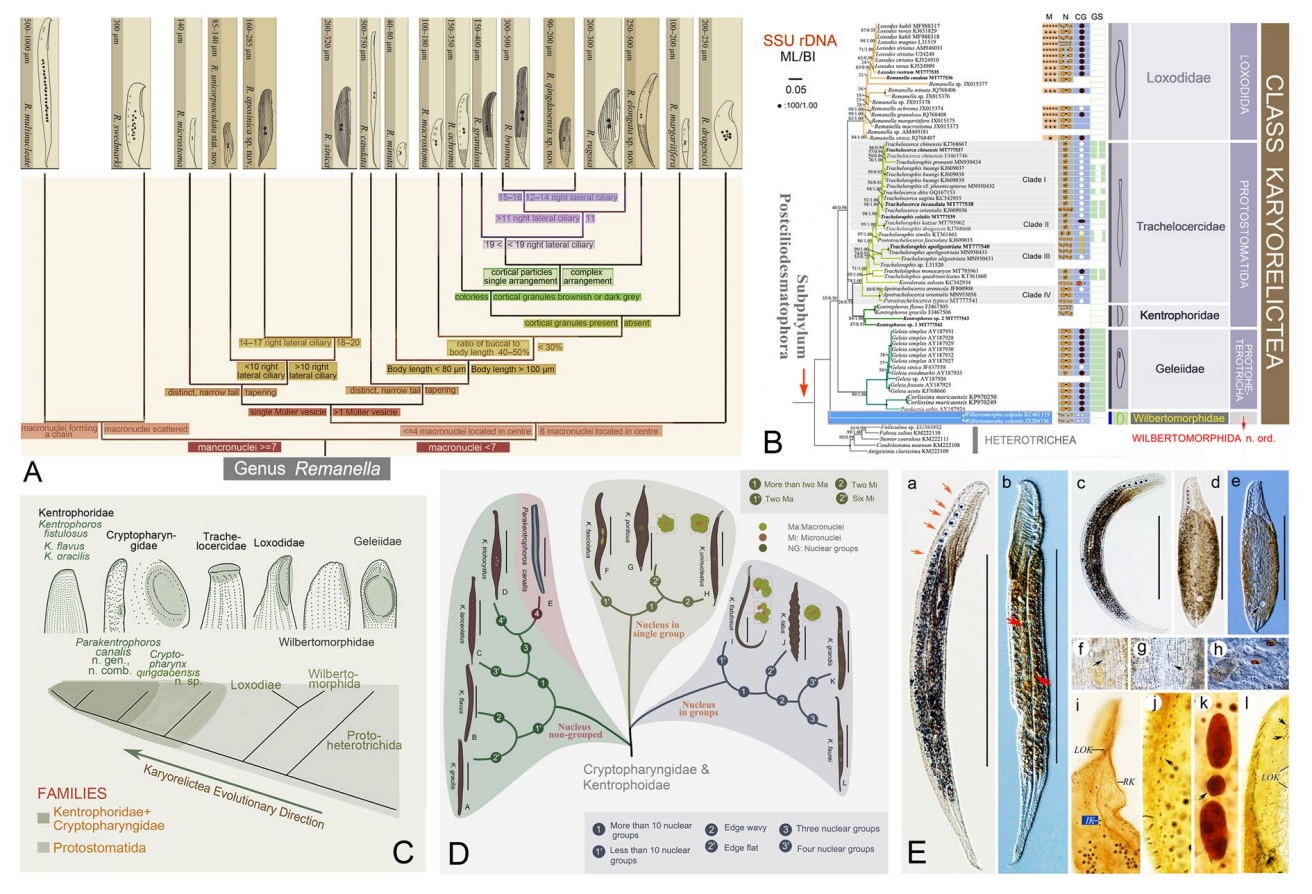
Morphology and phylogeny of karyorelictean ciliates. **A** Updated taxonomic key of the genus *Remanella* (Ma et al. 2022b). **B** Phylogenetic relationships focusing on karyorelicteans (Ma et al. 2022a). **C** Systematic classification and cladogram illustrating hypothesized evolutionary pathways in karyorelicteans (Ma et al. 2025). **D** Diagnostic key for recognized species of *Kentrophoros* and *Parakentrophoros* (Ma et al. 2025). **E** Morphological characteristics of *Remanella* (Ma et al. 2022b)

A detailed discussion regarding the phylogeny of karyorelicteans was recently published (Ma et al. 2022a). Based on this and previous analyses, the class Karyorelictea was confirmed to comprise four distinct orders and six families, all supported as distinct monophyletic lineages (Ma et al. 2022a; Xu et al. 2011b, c). As a new contribution, the OUC-group has characterized 118 sequences of key marker genes (69 SSU rDNA, 25 ITS-5.8S, and 24 LSU rDNA) covering four orders, six families and 14 genera, all of which have been submitted to the GenBank database (Gao et al. 2010; Ma et al. 2022a, b; Mazei et al. 2009; Xu et al. 2011b, c, d, 2013a, b, 2015, 2017; Ye et al. 2023). Phylogenetic analyses demonstrated that Protostomatida and Loxodida form sister clades, while Protoheterotrichida occupies a more basal position. Notably, Wilbertomorphida emerged as the earliest-diverging lineage, and was elevated to the order level (Ma et al. 2022a). These results provide a robust evolutionary framework for Karyorelictea, reconciling morphological and molecular evidence in a phylogenetic hypothesis (Ma et al. 2022a, b, 2025; Yan et al. 2016, 2017).

To date, the OUC-group has described one new order (Wilbertomorphida), one new family (Wilbertomorphidae), and four new genera (*Wilbertomorpha*, *Apotrachelocerca*, *Paratrachelocerca* and *Parakentrophoros*), as well as 21 new species within Karyorelictea, along with clarifying taxonomic confusions by establishing three new combinations (Ma et al. 2025; Xu et al. 2011b, 2014, 2017; Yan et al. 2016; Ye et al. 2023). However, compared to other groups of ciliates, karyorelicteans have been understudied. Their species-level diversity remains poorly understood, with many taxa being only partially described or awaiting investigation. In addition, many “known” forms lack the critical details (e.g., infraciliature and marker gene sequences) that are necessary for accurate species identification (Gao et al. 2010; Ma et al. 2025; Xu et al. 2017; Yan et al. 2016; Ye et al. 2023). Therefore, elucidating the alpha-taxonomy, particularly of psammophilic forms in intertidal habitats, is an immediate research goal. Moreover, the systematic position of many taxa is still undetermined. For example, *Parakentrophoros* is provisionally classified within the family Cryptopharyngidae, despite being the only species in this family to harbor symbiotic bacteria — a feature that is characteristic of the family Kentrophoridae. This suggests that *Parakentrophoros* may represent an intermediate lineage or even a novel family (Ma et al. 2025).

### Phylogenetic studies of the class Heterotrichea (Fig. 2)

The class Heterotrichea Stein, 1859, a “lower” taxon within the subphylum Postciliodesmatophora, represents an early-diverging branch of the phylum Ciliophora, alongside the Karyorelictea. Heterotrichs are characterized by a highly developed oral apparatus, comprising a paroral membrane and an adoral zone of membranelles (Lynn 2008). This morphological adaptation enables them to feed on bacteria, organic debris, other protists and even small metazoans (Foissner and Berger 1996). According to the currently accepted classification, this class comprises a single order containing approximately ten families (Shazib et al. 2014).

**Fig. 2:**
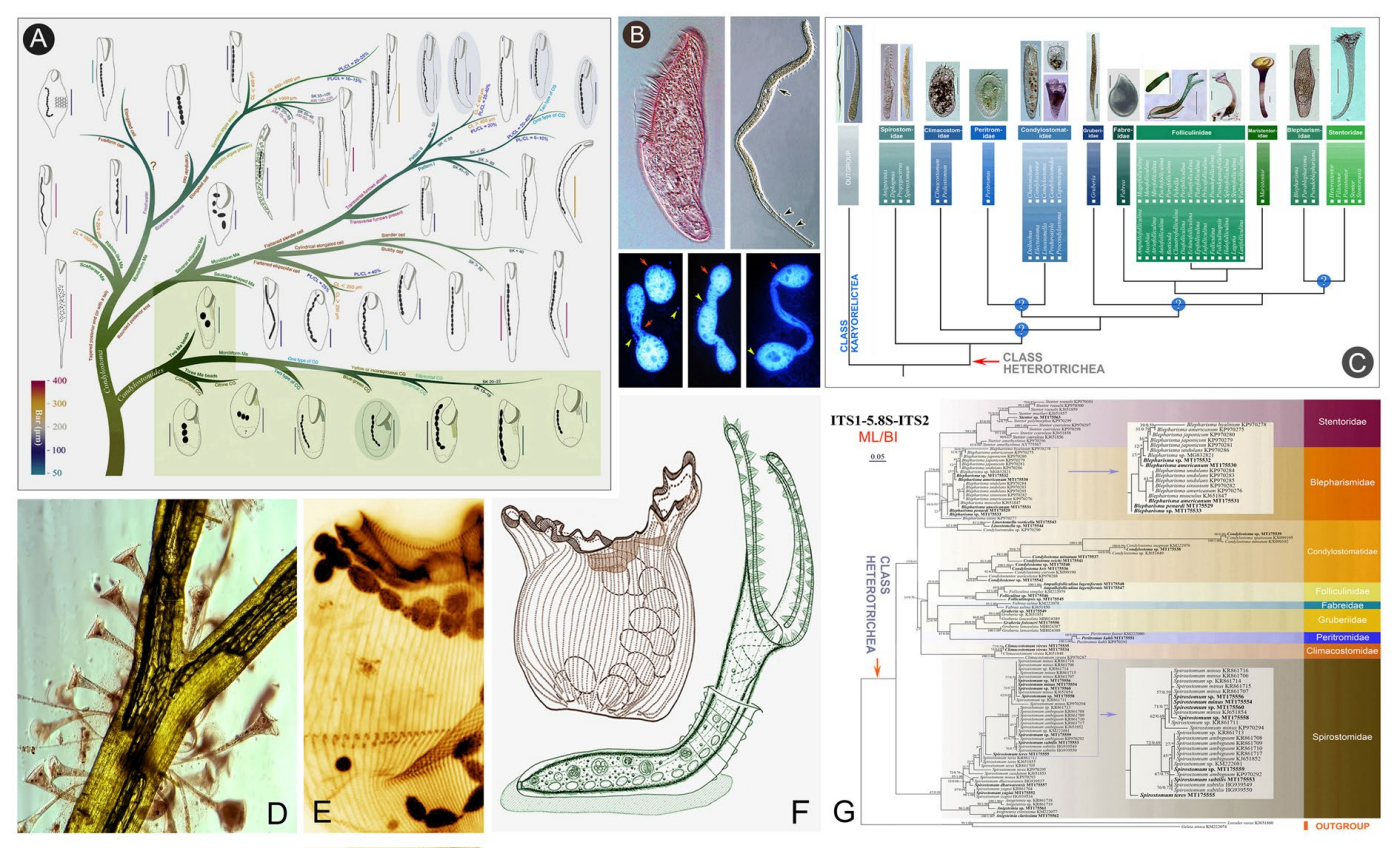
Morphology and phylogeny of the class Heterotrichea. **A** Illustrated key to valid *Condylostoma* and *Condylostomides* species (Chi et al. 2024b). **B** *Blepharisma stoltei* and its various macronuclei (Gong et al. In press), and *Spirostomum subtilis* (Chi et al. 2020). **C** Molecular cladogram of heterotrich families based on SSU rDNA phylogenetic analyses (Chi et al. 2021). **D** *Stentor* spp. **E** *Condylostentor auriculatus* (Chen et al. 2007). **F** Schematic drawings of a folliculinid species (Ye et al. In press). **G** Phylogenetic tree of the class Heterotrichea inferred from ITS1-5.8S-ITS2 gene sequences (Chi et al. 2021)

Recent reviews of the phylogenetic relationships of heterotrich groups have revealed that the family Peritromidae represents the earliest diverging branch within the class. In contrast, the families Stentoridae, Blepharismidae, Folliculinidae, Maristentoridae, and Fabreidae form the most recently diverging branches (Chi et al. 2021; Miao et al. 2009). These findings have led to the proposal of a new evolutionary hypothesis for the class Heterotrichea, which integrates both morphological and molecular data (Chi et al. 2021).

Additionally, a major revision of the family Folliculinidae standardized its terminology and identified six key morphological characters, leading to improved diagnoses of 33 nominal genera (Ye et al. 2022), as well as the reactivation of the genus *Diafolliculina* (Ye et al. 2021). A multigene phylogenetic analysis of the family Condylostomatidae, alongside a comprehensive review of 43 nominal species and approximately 130 populations from the type genus *Condylostoma* and its morphologically similar genus *Condylostomides*, concluded that the frontal membrane may have played a significant role in the evolutionary history of this family (Chen et al. 2019; Chi et al. 2024b).

Another significant outcome of these efforts is the substantial expansion of the genetic database for heterotrichs, with over 200 marker gene sequences covering nine families and 18 genera had been submitted to the GenBank database.

Our limited knowledge and understanding of the systematics of the heterotrichs is reflected in the re-identification, redefinition, and phylogenetic clarification of many groups that were previously considered “known”, particularly those with morphological similarities but insufficient molecular data. For instance, the highly species-rich family Blepharismidae lacks a comprehensive review, and its internal phylogenetic relationships inferred from the available sequences remain ambiguous. In addition, the presence of cryptic species within *Spirostomum minus* and *S. teres* (Chi et al. 2020, 2022b), as well as the recent discovery of cryptic species in *Condylostoma curvum* (Chi et al. 2024b), warrant further study. Finally, while the family-level boundaries within Heterotrichea are largely resolved, the phylogenetic positions of certain families, such as the Condylostomatidae and Climacostomidae, require further investigation.

### Phylogenetic studies of two poorly studied classes Prostomatea and Litostomatea (Figs. 3, 4)

The “lower group” of ciliates is largely a “melting pot” of mostly planktonic taxa scattered across phylogenetic branches (Song et al. 2009). However, they all exhibit a poorly differentiated somatic ciliature and a relatively simple oral structure (a plesiomorphic trait). This section focuses on two poorly studied classes, i.e., Prostomatea and Litostomatea, that were traditionally classified in the class Kinetofragminophora (Corliss 1979). The class Prostomatea currently comprises two orders (Prostomatida and Prorodontida) and about 11 families (Jiang et al. 2023). Free-living litostomateans are morphologically diverse and species-rich, encompassing over 10 orders and 20 families (Chi et al. 2024a; Vďačný et al. 2011).

**Fig. 3:**
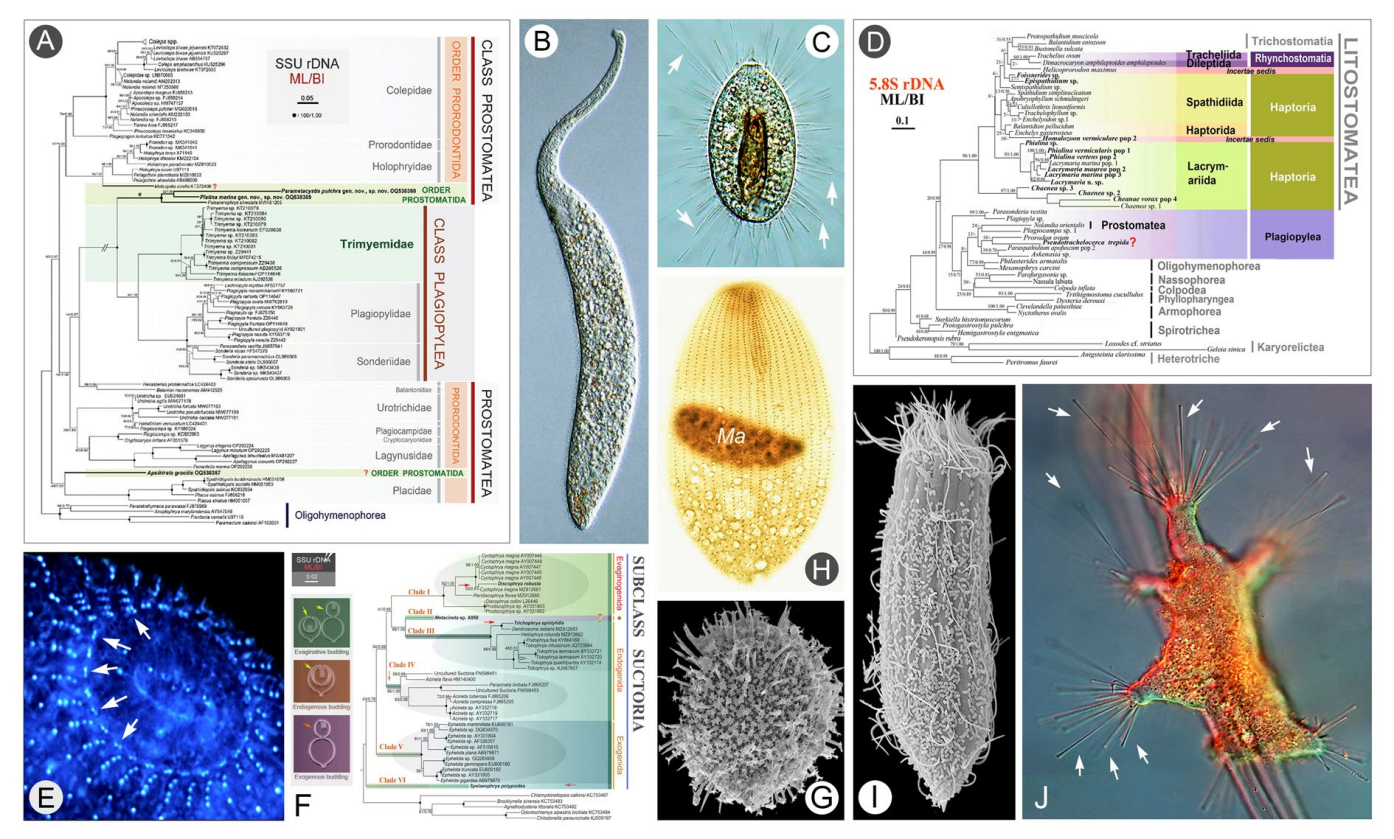
Morphology and phylogeny of the lower taxa. **A** Phylogenetic tree of the class Prostomatea inferred from SSU rDNA sequences (Jiang et al. 2024). **B** *Pseudomonilicaryon* sp. (Wang et al. 2022a). **C** *Actinobolina bivacuolata* (Chi et al. 2024a). **D** Phylogenetic tree of the class Litostomatea inferred from ITS1-5.8S-ITS2 gene sequences (Huang et al. 2018). **E** Infrastructure of anterior portion of *Coleps shanghaiensis* (Luo et al. 2024). **F** Phylogenetic tree of the subclass Suctoria inferred from SSU rDNA sequences (Ma et al. In press). **G** Cyst of *Balantidion pellucidum* (Chi et al. 2022a). **H** *Holophrya* sp. (Wang et al. 2022a). ** I** *Acropisthium mutabile* (Chi et al. 2022a). **J** *Trichophrya epistylidis* (Ma et al. In press)

**Fig. 4:**
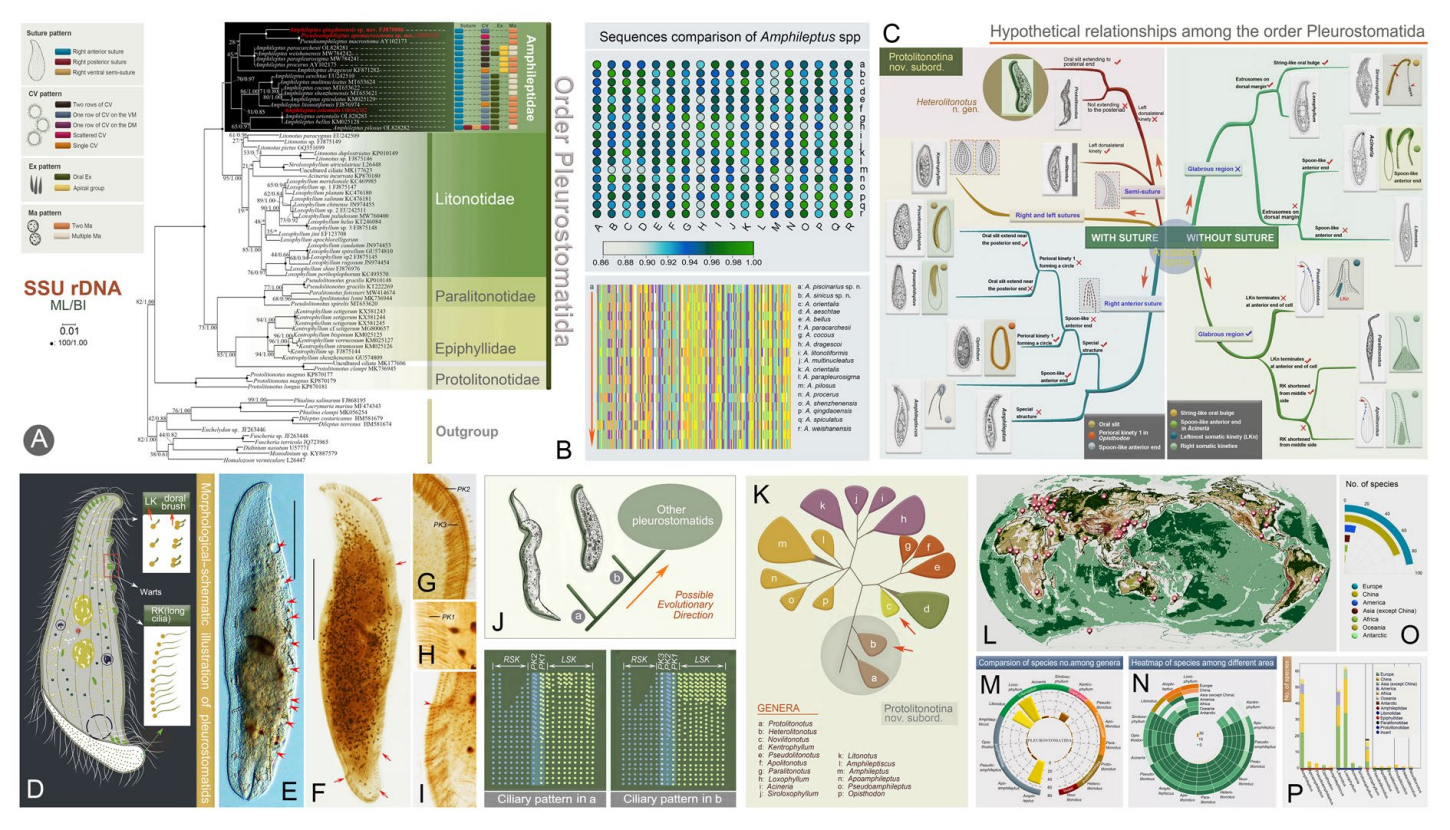
Morphology and phylogeny of pleurostomatids. **A** Phylogenetic tree inferred from SSU rDNA sequences of the order Pleurostomatida (Zhang et al. 2023a). **B** Sequence comparison of *Amphileptus* spp. (Zhang et al. 2024a).** C** Hypothetical relationships among pleurostomatids (Zhang et al. 2024c). **D** Morphology of a model pleurostomatid (Zhang et al. 2024c). **E**–**I** *Heterolitonotus rex* from life and after protargol staining (Zhang et al. 2024c). **J**, **K** Phylogenetic information and possible evolutionary trajectory of pleurostomatids (Zhang et al. 2024c). **L**–**P** Geographic distribution and genus-level diversity of pleurostomatids (Zhang et al. 2024c)

In studies of prostomatean ciliates over the past two decades, a total of six new genera have been established by the OUC-group: *Apocoleps*, *Apolagynus*, *Foissnerophrys*, *Parametacystis*, *Penardella*, and *Platina* (Chen et al. 2009; Jiang et al. 2021, 2023, 2024). Over 30 marker gene sequences have also been submitted to the GenBank database. The phylogenetic position of the family Apsiktratidae was elucidated for the first time, indicating a closer relationship to the order Prorodontida than to Prostomatida. This finding suggests that the oral basket may be a more phylogenetically informative feature than the brosse (Jiang et al. 2024). Additionally, the close relationship between Prostomatida and Trimyemidae (a family within the class Plagiopylea) was confirmed based on analyses of both their molecular phylogeny and the putative secondary structure of the V-9 region of SSU rDNA (Jiang et al. 2021, 2024). Furthermore, the validity of the genus *Levicoleps* has been called into question, with evidence suggesting that the presence or absence of armor spines is not a reliable diagnostic character for distinguishing between colepid genera (Lu et al. 2016; Luo et al. 2024). Despite these advances, the class Prostomatea remains poorly understood, particularly at the molecular level. Consequently, the internal phylogenetic relationships within this class, especially between its two orders (Prostomatida and Prorodontida), as well as its relationships with neighboring taxa such as Plagiopylea, require further investigation (Jiang et al. 2021, 2024).

Traditionally, free-living litostomateans were classified into two orders: Haptorida Corliss, 1974 and Pleurostomatida Schewiakoff, 1896 (Lynn 2008). Of these, the order Haptorida is one of the most morphologically diverse taxa, exhibiting a wide range of cell sizes and shapes, somatic ciliary patterns, and oral structures. This has led to persistent conflicts between morphological classifications and molecular phylogenies (Vďačný et al. 2011). Recent faunal studies of “traditional haptorids” have generated more than 80 sequences of marker genes from ten families, all of which have been submitted to the GenBank database. Based on these new data, phylogenetic analyses of the family Acropisthiidae were conducted for the first time. This led to the transfer of the historically ambiguous genus *Balantidion* into this family, a result supported by both morphological and molecular data (Chi et al. 2022a). Similarly, the phylogenetic position of the family Pseudotrachelocercidae was clarified, revealing that it does not belong to the class Litostomatea and should be regarded as *incertae sedis* within the subphylum Intramacronucleatea (Huang et al. 2018). Phylogenetic analyses also supported the exclusion of the genera *Cyclotrichium* and *Paraspathidium* from the class Litostomatea (Zhang et al. 2012). Moreover, a revision of the family Actinobolinidae revealed a highly divergent sister branch, emphasizing the significant morphological diversity within the order Spathidiida (Chi et al. 2024a).

The order Pleurostomatida is a distinct raptorial group within the class Litostomatea. Pleurostomatids are characterized by a laterally compressed body with a flattened, elongated oral region along the ventral margin. The cilia on the left and right sides are highly specialized (Lynn 2008). Members of this order are found all over the world and feed on flagellates, other ciliates, and even small metazoans such as rotifers (Foissner et al. 1995). Recent studies have indicated that this order comprises two suborders, five families, and 16 genera (Zhang et al. 2024c).

Phylogenetic analyses consistently support the monophyly of this order (Gao et al. 2008; Huang et al. 2018; Zhang et al. 2012, 2023a). Building upon recent advances in the availability of morphological and molecular data, Pleurostomatida has been reclassified into two suborders: Protolitonotina Zhang et al., 2024 and Amphileptina Jankowski, 1967. The suborder Protolitonotina, comprising the genera *Protolitonotus* Wu et al., 2017 and *Heterolitonotus* Zhang et al., 2024, represents the ancestral pleurostomatid lineage (Zhang et al. 2024c). A comprehensive, multi-gene phylogenetic analysis of pleurostomatids confirmed that the genus *Protolitonotus* Wu et al., 2017 forms a distinct lineage and family (Protolitonotidae). This family is characterized by the right somatic kineties forming a semi-suture near the dorsal margin (Wu et al. 2017).

Within the family Paralitonotidae Zhang et al., 2022, three genera — *Apolitonotus* Pan et al., 2020, *Pseudolitonotus* Wu et al., 2022, and *Paralitonotus* Zhang et al., 2022 — lack some anterior basal bodies in their right somatic kineties, forming a glabrous region. The development of this feature may have contributed to their divergence from the core pleurostomatids (Pan et al. 2020; Wu et al. 2022a; Zhang et al. 2022a).

An updated taxonomic scheme recognizing five monophyletic groups within Pleurostomatida has been proposed, and a hypothetical evolutionary trajectory of pleurostomatids has been inferred (Zhang et al. 2022a). Furthermore, a revised identification key covering all five families and 16 pleurostomatid genera has also been developed (Zhang et al. 2024c). Over the past two decades, more than 70 marker gene sequences covering five families and 13 genera have been deposited in the GenBank database. Thus, our research has filled critical gaps in molecular data for this group, yielding a wealth of new findings.

Despite the extremely high diversity of litostomateans, only a few common species have been described in detail, and molecular data are available for fewer than 10% of these species. Consequently, our understanding of their true phylogenetic relationships remains limited (Chi et al. 2024a). Even for some “well-known” orders (e.g., Spathidiida and Haptorida), their proper placement within systematic trees remains unresolved. In addition, taking the order Pleurostomatida as an example, the phylogenetic relationships within the family Amphileptidae Bütschli, 1889 remain unclear. The positions of genera, such as *Opisthodon* Stein, 1859 and *Amphileptiscus* Song and Bradbury, 1998, require further investigation. Furthermore, the paraphyly of the genus *Amphileptus* Ehrenberg, 1830 awaits confirmation using more extensive data and analyses. Therefore, future studies should prioritize broader sampling to cover more groups and help resolve the systematics of these little-known taxa. In addition, constructing a robust and comprehensive phylogenetic framework using multiple molecular markers should be the next task.

### Phylogenetic studies of obligate anaerobic ciliates (Fig. 5)

Obligate anaerobic ciliates are characterized by their symbiotic associations with prokaryotes and the presence of mitochondrion-related organelles (MROs). They inhabit diverse anoxic or hypoxic environments (Fenchel and Finlay 2018; Feng et al. 2020; Hao et al. 2025; Hu 2014; Rotterová et al. 2020, 2022; Xu et al. 2025). There are currently six classes that exclusively comprise obligate anaerobic ciliates: Armophorea Lynn, 2004, Plagiopylea Small and Lynn, 1985, Cariacotrichea Orsi et al., 2012, Odontostomatea Fernandes et al., 2018, Muranotrichea Rotterová et al., 2020, and Parablepharismea Rotterová et al., 2020 (Fernandes et al. 2018; Lynn 2008; Orsi et al. 2012; Rotterová et al. 2020). Armophorea and Plagiopylea are the most species-rich of these taxa and both lack morphological apomorphies; they are riboclasses that are defined solely by SSU rDNA sequences (Lynn 2008).

**Fig. 5:**
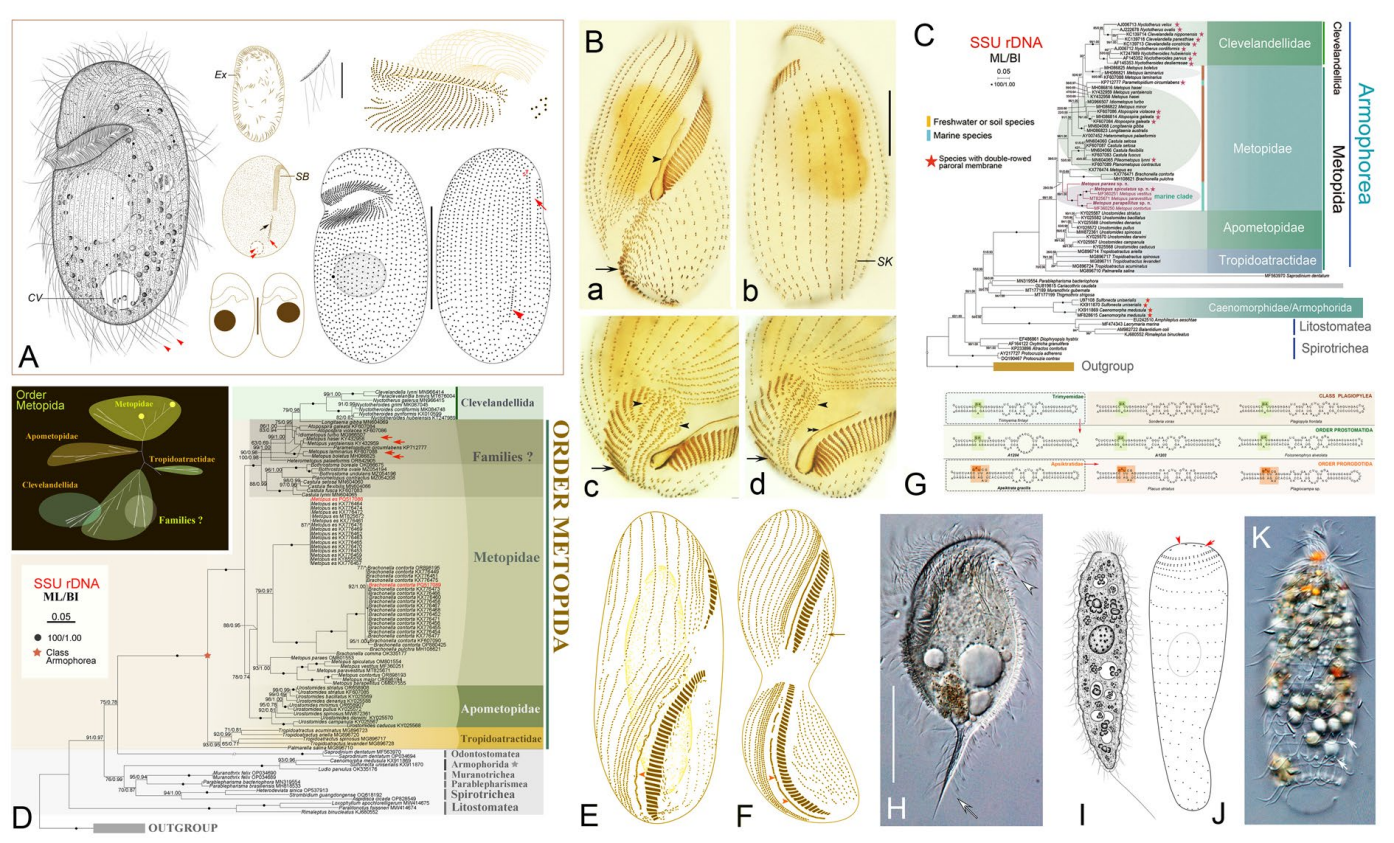
Morphology and phylogeny of obligate anaerobic ciliates. **A** *Plagiopyla rariseta* (Li et al. 2023b). **B, E, F** Morphogenesis of *Metopus es* (a, b in B; E, F) and *Brachonella contorta* (c, d in B) (Feng et al. 2025). **C, D** Phylogenetic relationships of Armophorea based on SSU rDNA sequences (C from Zhuang et al. 2022; D from Feng et al. 2025). **G** Secondary structure of V9 region of SSU rDNA sequences of some representative plagiopyleans and prostomateans (Jiang et al. 2024). **H** *Metopus spiculatus* (Zhuang et al. 2022). **I**−**K** *Platina marina* (I, J) and *Parametacystis pulchra* (K) (Jiang et al. 2024)

Due to the lack of molecular data, few studies on the phylogeny of these groups had been conducted by the early twenty-first century (Lynn and Wright 2013; Vďačný et al. 2010). Recently, 18 SSU rDNA sequences from 16 species and seven genera within the non-monophyletic class Armophorea were deposited in the GenBank database by the OUC-group. All available data support the assertion that the genus *Metopus* represents an independent evolutionary lineage, with habitat adaptation potentially driving divergence within the genus (Li et al. 2021c; Zhuang et al. 2022). However, several long-standing issues remain unresolved. For instance, the systematic status of the order Armophorida and whether this taxon should be elevated to class level, remains uncertain (Li et al. 2023a). Furthermore, the possibility that most *Metopus* species should be assigned to different genera or families, leaving only the type species *Metopus es* and its closely related congeners as true *Metopus*, requires further investigation (Bourland et al. 2020; Feng et al. 2025; Zhuang et al. 2022).

The class Plagiopylea is widely recognized as comprising one order containing three families: Plagiopylidae, Sonderiidae, and Trimyemidae (Fernandes et al. 2018; Lynn 2008). Through single-gene phylogenetic analyses, we assessed the phylogenetic position of plagiopyleans and confirmed the monophyly of the families Plagiopylidae and Trimyemidae (Li et al. 2021b, 2022, 2023b, c, 2024a; Nitla et al. 2019). All nominal sonderiids are divided into two clades, comprising the genera *Parasonderia* and *Sonderia*, respectively (Li et al. 2024a). Notably, all currently available SSU rDNA sequences of *Parasonderia* have been derived from published studies of the OUC-group (Li et al. 2024a; Xu et al. 2013c).

As is commonly observed in other ciliate groups, piecemeal studies have hindered systematic analyses, an issue that is particularly pronounced in anaerobic lineages. Many order- and family-level taxa lack precise phylogenetic placement, which is a key focus of our near-term research efforts. Based on integrative analyses of molecular and morphological data, a hypothesis has been proposed suggesting that the shape of the oral opening is a reliable feature for distinguishing *Parasonderia* species (Li et al. 2024a). This hypothesis also applies to the two clades of the family Plagiopylidae (Li et al. 2023b). Furthermore, we have updated the taxonomic key for valid Trimyemidae species and evaluated their key morphological traits based on phylogenetic analyses (Li et al. 2023b, c). However, further integrative studies incorporating multiple marker genes or omic information are required to clarify the internal relationships of Plagiopylea. This is particularly important given the low bootstrap value supporting the monophyly of Sonderiidae, and the lack of informative morphological features corresponding to the phylogenetic position of Trimyemidae.

### Phylogenetic studies of the subclass Cyrtophoria (Fig. 6)

Ciliates belonging to the subclass Cyrtophoria form a specialized group in terms of their morphology. Cyrtophorians are typically either dorsoventrally flattened or laterally compressed, and possess a well-developed cytopharyngeal basket. Additionally, their cilia are confined to the ventral side and their macronucleus is heteromerous. According to Lynn (2008), the subclass Cyrtophoria comprises two orders: Chlamydodontida and Dysteriida. Most species in the order Dysteriida have a laterally compressed body and the presence of either a non-ciliated adhesive region or a podite. In contrast, members of the order Chlamydodontida are characterized by a dorsoventrally flattened body and an absence of the podite (Deroux 1974, 1976a, b; Lynn 2008; Qu et al. 2022).

**Fig. 6:**
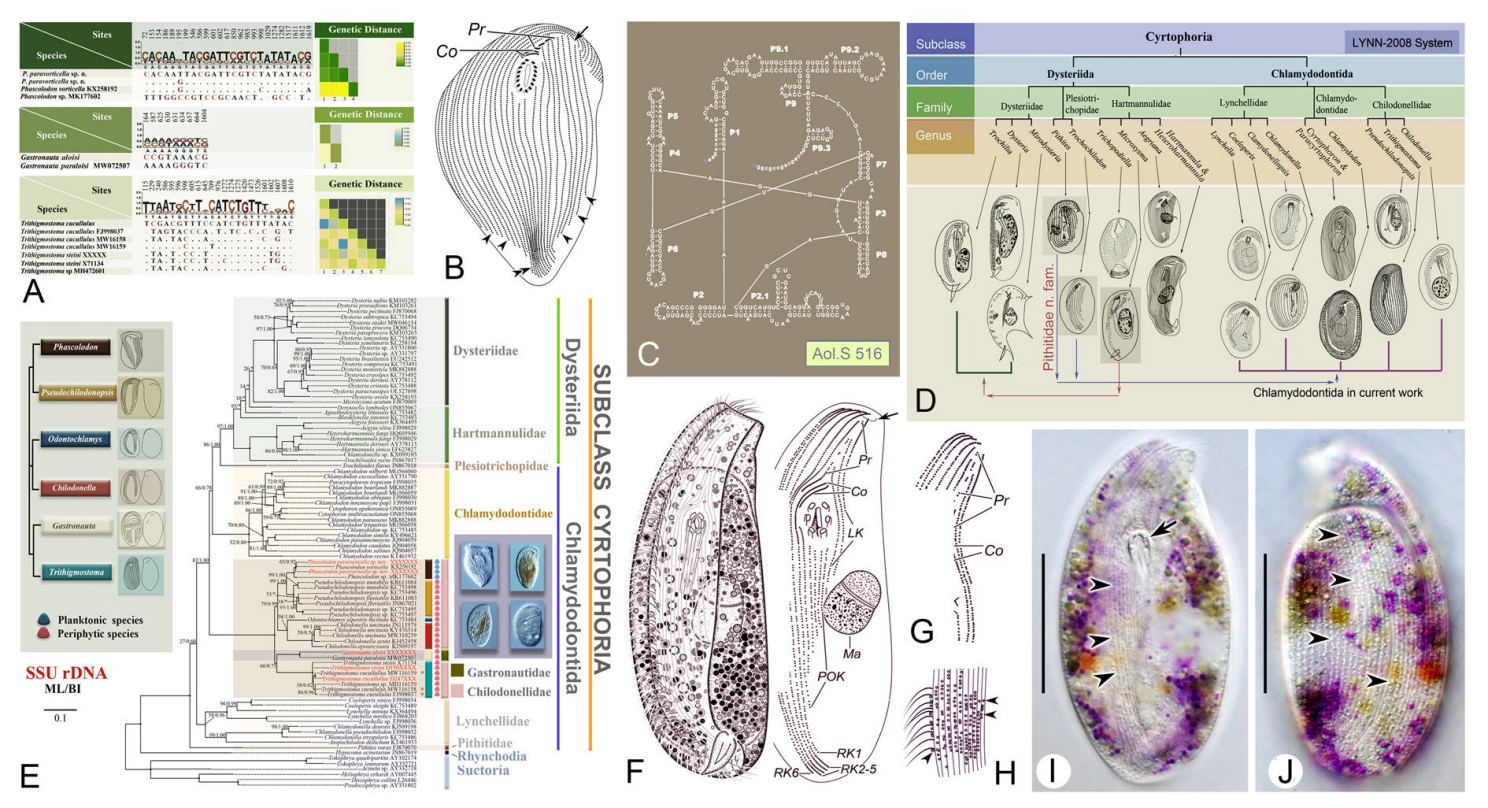
Morphology and phylogeny of the subclass Cyrtophoria. **A** Nucleotide differences and genetic distances based on SSU rDNA sequences (Zhang et al. 2024b). **B** Ventral view infraciliature of *Cyrtophoron apsheronica* (Wang et al. 2023). **C** Secondary structure of the introns in the SSU rDNA of *Aegyria oliva* (Gao et al. 2012). **D** Schematic diagrams of some representative morphospecies (Gao et al. 2012). **E** The maximum likelihood (ML) and Bayesian inference (BI) trees inferred from SSU rDNA sequences (Zhang et al. 2024b). **F-J** *Derouxella lembodes* from life and after protargol staining (Wang et al. 2023)

Over the past two decades, we have conducted in-depth, molecular-based taxonomic research, resulting in a total of over 140 marker gene sequences being submitted to the GenBank database, covering seven families and 28 genera. This contribution has not only expanded the gene database of cyrtophorid ciliates but also provided a foundation for studying the phylogenetic relationships among Cyrtophoria (Chen et al. 2016; Gao et al. 2012; Wang et al. 2017a). The family Lynchellidae is the earliest diverging branch of the Cyrtophoria and is most closely related to the ancestral cyrtophorids. In stark contrast, the families Dysteriidae and Kyaroikeidae are located within the crown group of the subclass Cyrtophoria, and represent the most recently diverged branch (Pan et al. 2017; Qu et al. 2022; Wang et al. 2025).

The most morphologically specialized groups, Chilodonellidae and Gastronautidae, evolved independently in freshwater environments and together constitute the longest evolutionary branch (Qu et al. 2022; Zhang et al. 2024b). The genera within the family Chlamydodontidae display virtually identical ciliary patterns. This is also reflected in the SSU rDNA trees, where they form a fully supported clade (Qu et al. 2018a, b).

In addition, based on its unique oral apparatus and position within the phylogenetic tree, we have established the family Pithitidae for the species *Pithites vorax* (Gao et al. 2012). We consider Hartmannulidae to be the earliest diverging branch within the order Dysteriida. Moreover, we identified a novel species, *Derouxella lembodes*, that exhibits close phylogenetic relationships with both Hartmannulidae and Dysteriidae. It is currently classified as *incertae sedis* within Dysteriida (Wang et al. 2023).

In terms of its phylogeny, Cyrtophoria is probably the best-resolved subclass within the phylum Ciliophora. The predominant issue, however, remains the paucity of molecular data. Only 40% of known species have available marker gene sequences, and a small number of these lack morphological data to underpin their molecular phylogeny. Consequently, current phylogenetic trees do not always accurately reflect evolutionary relationships. For instance, the systematic positions of the families Pithitidae and Plesiotrichopidae, as well as several species, including *Derouxella lembodes* and *Brooklynella sinensis*, remain uncertain (Qu et al. 2022). Key morphological features such as cross-striated bands and finger-like tentacles have not been adequately reflected in their evolutionary relationships on the phylogenetic tree (Wang et al. 2023, 2025). Further analysis of morphological and molecular phylogenetic data is therefore necessary.

### Phylogenetic studies of the subclass Scuticociliatia (Fig. 7)

Scuticociliates are characterized by the presence of a scutica (a transient kinetosomal structure or organelle that is present during stomatogenesis). They are one of the most species-rich groups of ciliates that occupy various aquatic and terrestrial habitats, either free-living or as parasites of aquatic animals (Poláková et al. 2023; Song et al. 2003; Zhang and Vďačný 2024). Their small size and similar morphological characteristics often complicate taxonomic and ecological studies (Fan et al. 2011; Pan et al. 2013). The subclass Scuticociliatia Small, 1967 is currently recognized as comprising three orders: Philasterida Small, 1967, Pleuronematida Fauré-Fremiet in Corliss, 1956, and Loxocephalida Jankowski, 1980 (Gao et al. 2016; Lynn 2008). However, the phylogenetic relationships within the subclass Scuticociliatia remain largely unresolved due to insufficient molecular data, as well as conflicts between molecular and morphological interpretations (Foissner et al. 2009; Lynn and Strüder-Kypke 2005).

**Fig. 7:**
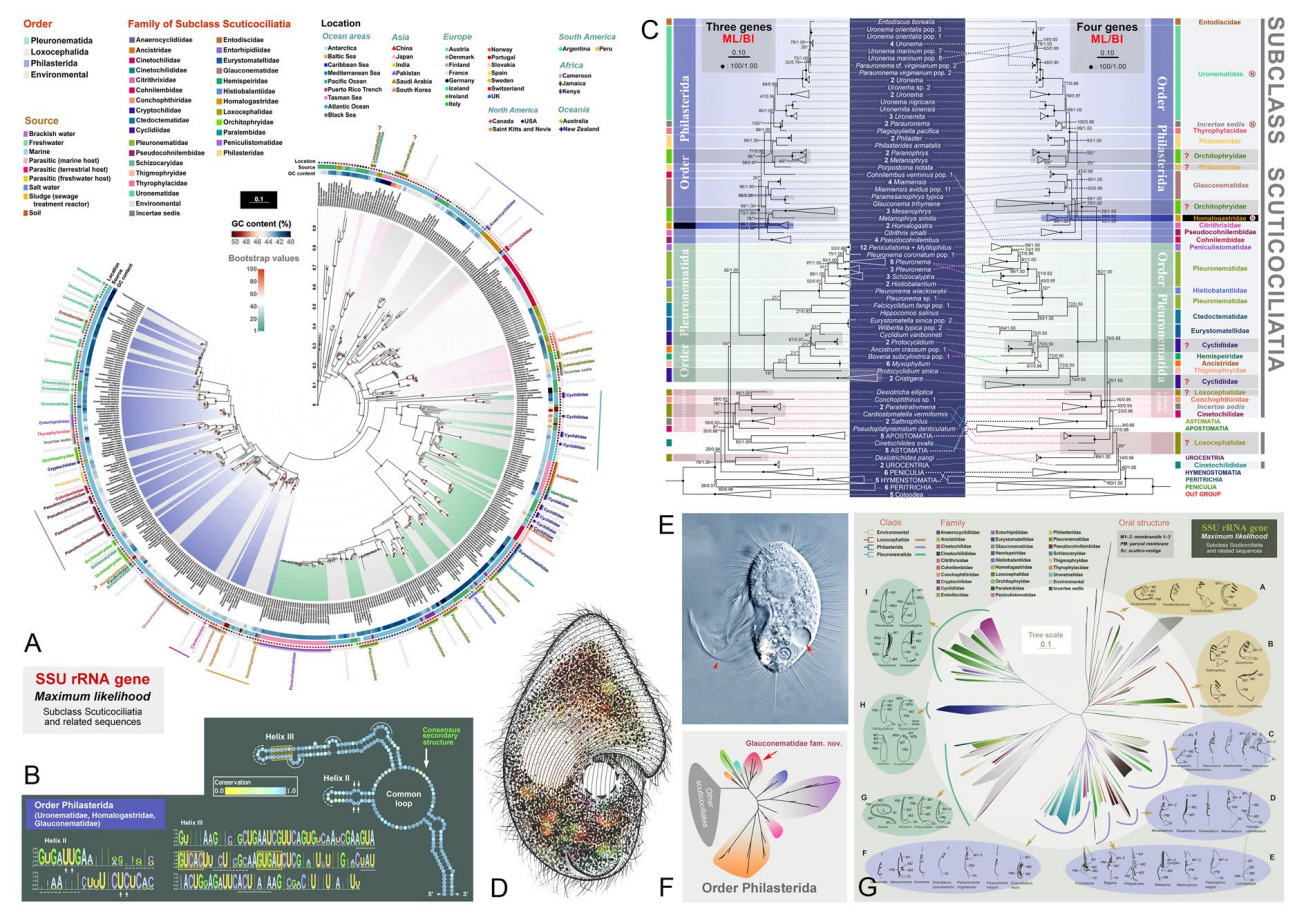
Morphology and phylogeny of the subclass Scuticociliatia. **A, C** Maximum likelihood (ML) tree based on SSU rDNA sequences (Liu et al. 2025). **B** Consensus secondary structure of putative ITS2 region of taxa from Uronematidae, Homalogastridae, and Glauconematidae of the order Philasterida (Liu et al. 2025). **D** *Conchophthirus lamellidens* (Li et al. 2024c). **E** *Pleuronema parasetigerum* (Li et al. 2024b). **F** Unrooted ML tree based on SSU rDNA sequences for selected families in the order Philasterida (Li et al. 2024b). **G** Collapsed ML tree of Scuticociliatia based on SSU rDNA sequences with schematic oral structure diagrams of some representatives (Liu et al. 2025)

Over the past three decades, we have expanded the taxonomic sampling of scuticociliates and characterized 278 marker gene sequences, including 101 nuclear SSU rDNA, 58 ITS-5.8S rDNA, 44 LSU rDNA, 29 mitochondrial SSU rDNA and 46 mitochondrial cytochrome c oxidase subunit 1 (COI) genes. Phylogenetic analyses based on single-gene and concatenated data have been carried out to elucidate the phylogenetic relationships within the subclass Scuticociliatia. Some of these findings have been summarized in Gao et al. (2016); therefore, this review focuses exclusively on studies since 2017.

Since 2017, the OUC-group has identified and investigated 23 species (including 14 new species), covering 13 genera (including one new genus *Citrithrix*) (e.g., Liu et al. 2022). Based on updated morphological data and molecular phylogenetic analyses, three new families (Citrithrixidae, Glauconematidae, and Homalogastridae) have been established (Li et al. 2024b; Liu et al. 2020, 2025). The family Glauconematidae was established to include the genera *Glauconema*, *Miamiensis*, *Paramesanophrys*, and *Anophryoides*, which are characterized by their polymorphic life cycle (Li et al. 2024b). Both the families Citrithrixidae and Homalogastridae are monogeneric, distinguished by their unique morphological and molecular characteristics (Liu et al. 2020, 2025). Furthermore, we applied the first mitochondrial DNA-based phylogenetic framework to Scuticociliatia, establishing new molecular markers for this taxonomically challenging group and demonstrating the utility of mtSSU-rRNA V4 region secondary structures for discriminating closely related taxa (Zhang et al. 2019). Moreover, the most comprehensive systematic analysis to date, incorporating extensive sampling coverage (including 147 environmental SSU rDNA sequences), has elucidated a more robust phylogenetic framework for Scuticociliatia. Based on this framework, the evolutionary diversification of the oral structure across the subclass has been summarized and discussed (Liu et al. 2025).

Despite recent advances, significant challenges persist in scuticociliate phylogeny and systematics. For example, both nuclear and mitochondrial phylogenetic analyses have revealed that the order Loxocephalida is polyphyletic and represents some divergent and intermediate lineages between the subclasses Scuticociliatia and Hymenostomatia (Zhang et al. 2019). Additionally, unlike the free-living morphotypes, there is limited information on the infraciliature and molecular systematics of parasitic taxa. Therefore, comprehensive taxonomic sampling, especially the type species of loxocephalids and parasitic species, combined with phylogenomic approaches, is critically needed to establish a robust classification framework for scuticociliates.

### Phylogenetic studies of the peritrich order Sessilida (Figs. 8, 9)

The order Sessilida is the dominant group of peritrichs, comprising about 800 nominal species. It is one of the most species-rich ciliate groups in aquatic ecosystems (Foissner et al. 1992; Lynn 2008; Wang et al. 2022a). Most sessilids exhibit a biphasic life cycle, alternating between sessile trophonts and free-swimming telotrochs, though certain species are exclusively pelagic (Chen et al. 2022; Wu et al. 2020, 2024). Traditional classification schemes recognize 14 families within this order, primarily delineated by morphological characters such as the solitary or colonial lifestyle, presence or absence of a stalk, and stalk with or without a spasmoneme (Lynn 2008; Li et al. 2008b; Wu et al. 2020, 2021a, b, 2022c). However, our integrated morphological-molecular analyses reveal persistent ambiguities in family-level boundaries. Current diagnostic characters of sessilids, predominantly based on living morphology, appear evolutionarily labile and prone to homoplasy (Wang et al. 2022b). The families Epistylididae and Operculariidae are among the most problematic groups, species of which are scattered among several different clades in phylogenetic trees.

**Fig. 8:**
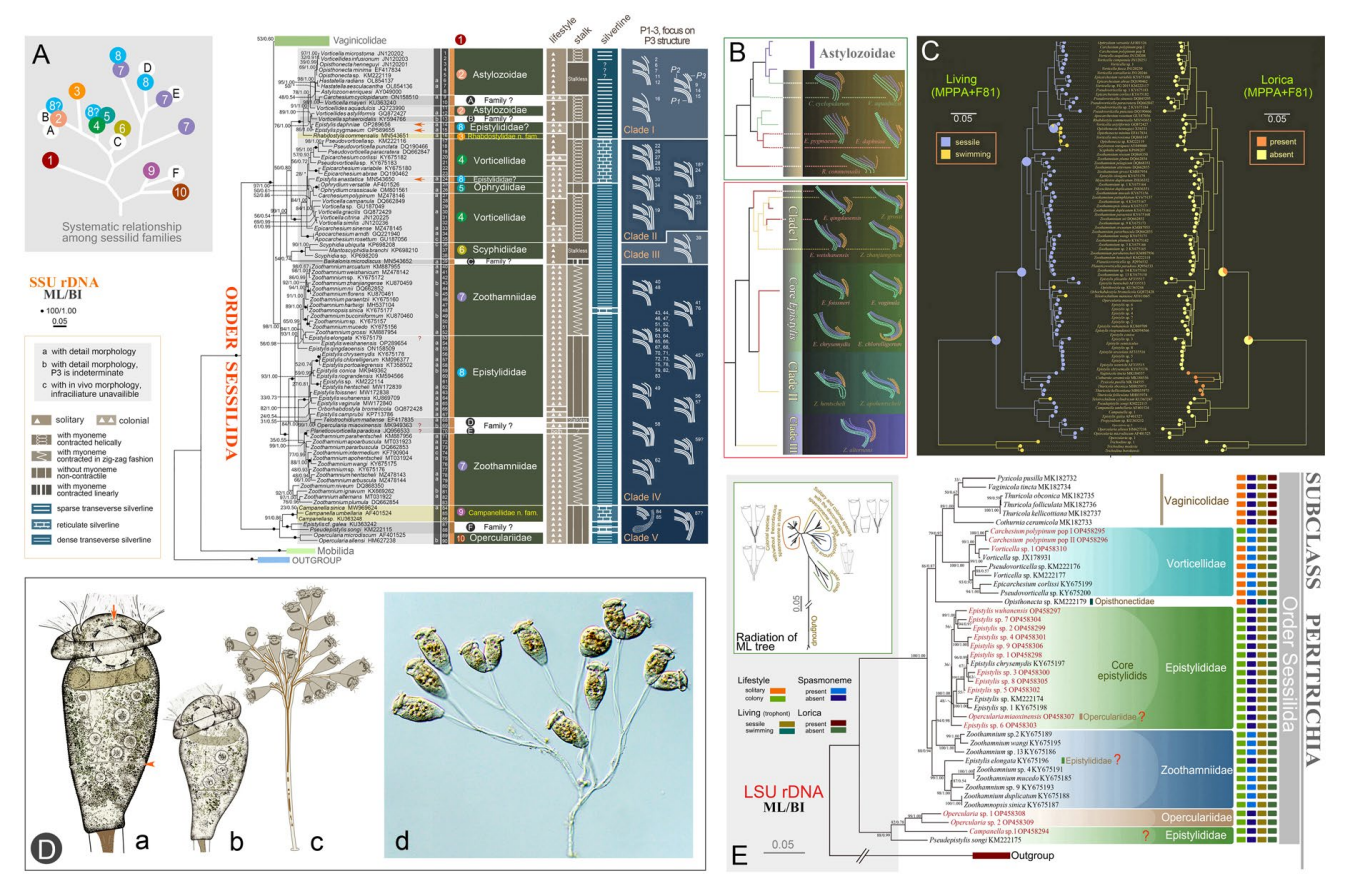
Morphology and phylogeny of the order Sessilida. **A, B** Phylogenetic tree based on SSU rDNA sequences (Lu et al. 2023; Wu et al. 2023). **C** Ancestral living habit and lorica reconstruction for sessilids based on the SSU rDNA ML tree (Wang et al. 2022b). **D** Representative species of sessilids (Wu et al. 2024). a, b: Extended zooids of *Zoothamnium procerius*, arrow indicates the contractile vacuole, arrowheads indicate the trochal band. c: A typical colony of *Zoothamnium procerius*. d: A typical colony of *Epicarchesium granulatum*. **E** Phylogenetic tree based on LSU rDNA sequences (Wang et al. 2022b)

**Fig. 9:**
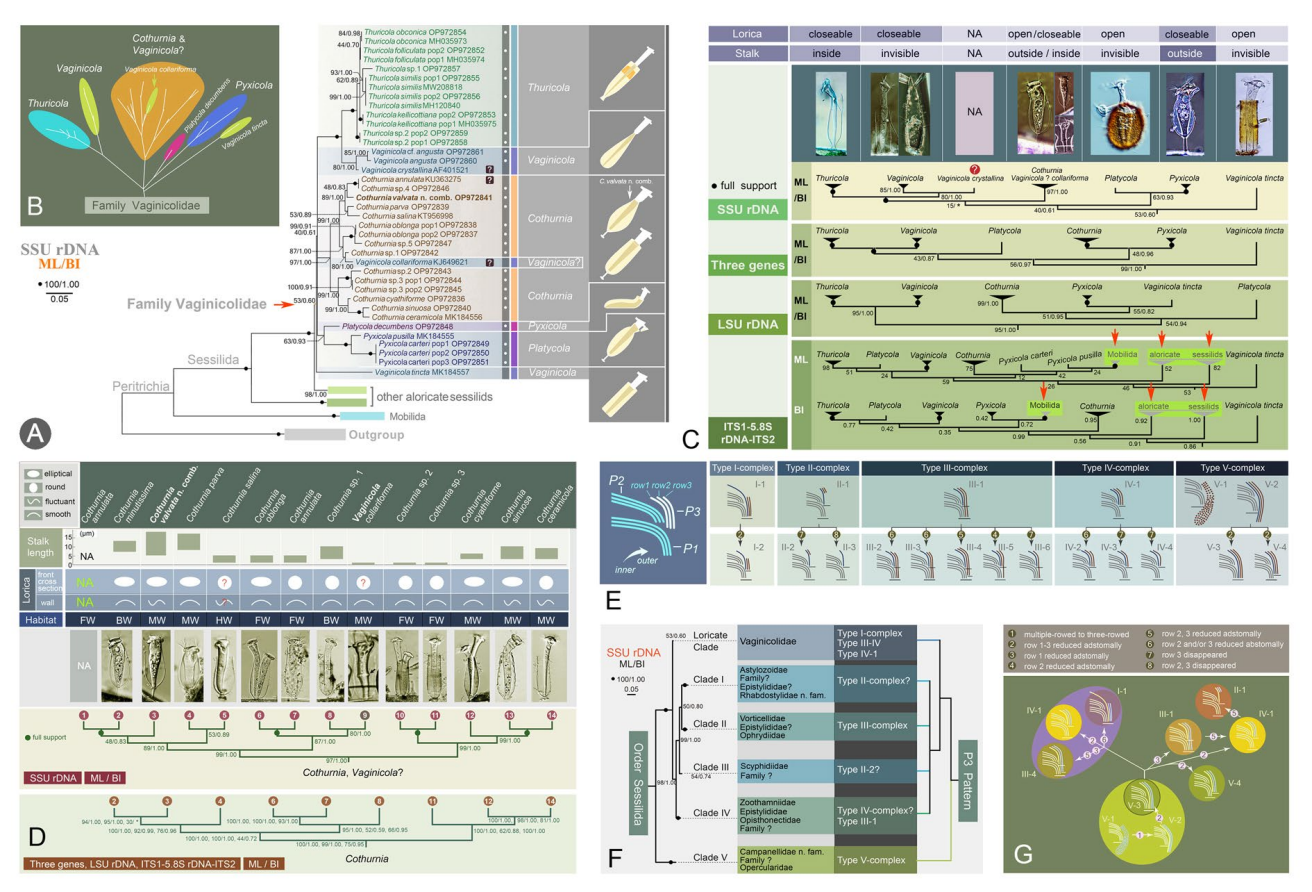
Phylogenetic studies of the family Vaginicolidae (Lu et al. 2023). **A, B** SSU rDNA-based trees. **C** Comparison of trees inferred from different datasets. **D** Topology within the clade including all *Cothurnia* species, with habitat and morphological data mapped. **E** Summary patterns of infundibular polykineties 3 (P3). **F** Correlation between the SSU rDNA-based phylogeny and P3 structure. **G** Putative evolutionary process of P3

To address these taxonomic inconsistencies, the OUC-group has: (1) elevated two genera, *Rhabdostyla* and *Campanella,* to family level, i.e., Rhabdostylidae and Campanellidae (Lu et al. 2023; Wang et al. 2021d); (2) transferred an epistylidid-like species to a newly established genus, *Parapiosoma* (Wu et al. 2023); (3) verified the loss of the stalk spasmoneme during the evolution of core Epistylididae from zoothamniid-like ancestors (Lu et al. 2023; Wang et al. 2022b); (4) proposed that epistylidids nested in the basal sessilid clade might belong to a new family-level taxon (Wang et al. 2022b; Wu et al. 2023); (5) suggested that the colonial operculariids, with a reduced epistomial disc and which invariably cluster with core epistylidids may represent a distinct family (Wang et al. 2022c); (6) indicated that a vorticellid, i.e. *Baikalonis microdiscus*, should be assigned to an independent lineage (Lu et al. 2020). Crucially, comparative analysis of the structure of infundibular polykinety 3 (P3) is more taxonomically informative than gross morphological traits, providing a useful character for reflecting evolutionary relationships among sessilids (Lu et al. 2020; Wu et al. 2022b).

The family Vaginicolidae de Fromentel, 1874 is a diverse group comprising nearly 200 nominal species, accounting for over two-thirds of reported loricate peritrichs (Mayen-Estrada and Clamp 2016; Lu et al. 2023). Vaginicolidae receives little attention compared to non-loricate peritrichs, as evidenced by the fact that nearly 90% of species lack both molecular data and modern-standard morphological data. This makes it difficult to determine evolutionary relationships within the family.

In recent years, the OUC-group has investigated 32 populations, representing 23 species from six genera: *Cothurnia*, *Cothurniopsis*, *Platycola*, *Pyxicola*, *Thuricola*, and *Vaginicola*. Based on newly obtained molecular and morphological data (32 SSU rDNA, 28 ITS-5.8S rDNA, and 28 LSU rDNA have been submitted to GenBank), phylogenetic analyses of Vaginicolidae have been conducted (Lu et al. 2018, 2019, 2023). The main findings were as follows: (1) the monophyly of the family Vaginicolidae, and of the genera *Thuricola* and *Pyxicola,* is supported. This implies that vaginicolids probably evolved independently, and that the closable structure (valve) in the lorica of *Thuricola,* and the operculum of *Pyxicola,* are apomorphies; (2) the monotypic *Cothurniopsis* should be synonymized with *Cothurnia*, as it consistently nested within *Cothurnia* in molecular phylogenetic trees; (3) *Vaginicola collariforma,* which nests deep among *Cothurnia* species, is probably a misidentified *Cothurnia* species. If so, *Cothurnia* would become monophyletic after synonymizing *Cothurniopsis* with it; (4) *Vaginicola* is non-monophyletic, which discredits the morphological definition of this genus and necessitates a re-evaluation of the diagnostic character (possession of a stalk outside the lorica) used to separate *Vaginicola* from *Cothurnia*; (5) SSU rDNA is highly conserved in Vaginicolidae, with little or no intraspecific variation.

As one of the most species-rich ciliate groups, our understanding of sessilid systematics faces four major challenges: (1) severe taxonomic chaos, with conflicting classification schemes; (2) unverified species identities, with a lack of reliable diagnostic characters for most known isolates; (3) undiscovered diversity, with potentially hundreds of cryptic species awaiting detection; (4) a critical molecular deficit, with genetic data available for less than 20% of known species. To date, we have deposited about 200 rDNA sequences in the GenBank database, each supported by accurate species identification. Paradoxically, increased species sampling intensifies phylogenetic incongruence within Sessilida, particularly among Epistylididae, Zoothamniidae, and Vorticellidae (Wu et al. 2021a, 2022b, 2023, 2024). Nevertheless, our findings suggest that thorough taxonomic revisions or refinements within the order Sessilida remain premature, given the substantial number of unknown or rare species yet to be studied. Resolving these systematic ambiguities necessitates the integration of detailed morphological information and molecular datasets.

### Phylogenetic studies of the subclasses Oligotrichia and Choreotrichia (Fig. 10)

Oligotrichs (*sensu lato*), comprising the subclasses Oligotrichia and Choreotrichia, dominate the microzooplankton community. They are regarded as a crucial link within the microbial food web, playing pivotal roles in energy flow and element cycling in both marine and freshwater ecosystems (Pierce and Turner 1992). They are characterized by their globular to obconical cell bodies and the apically located adoral zone of membranelles. This combination of features significantly enhances their swimming ability, enabling them to adapt effectively to the planktonic lifestyle (Agatha and Strüder-Kypke 2014). The OUC-group began researching oligotrichs (*s.l.*) in 1999 (Song et al. 1999). To date, a total of 26 studies focusing on the phylogeny of oligotrichs (*s.l.*) have been published. Prior to 2020, research primarily focused on aloricate oligotrichs (e.g., Liu et al. 2011a, 2016; Song et al. 2023). Since 2020, however, several tintinnids have been described or redescribed, including details of their infraciliature (e.g., Bai et al. 2020a; Wang et al. 2021c).

**Fig. 10:**
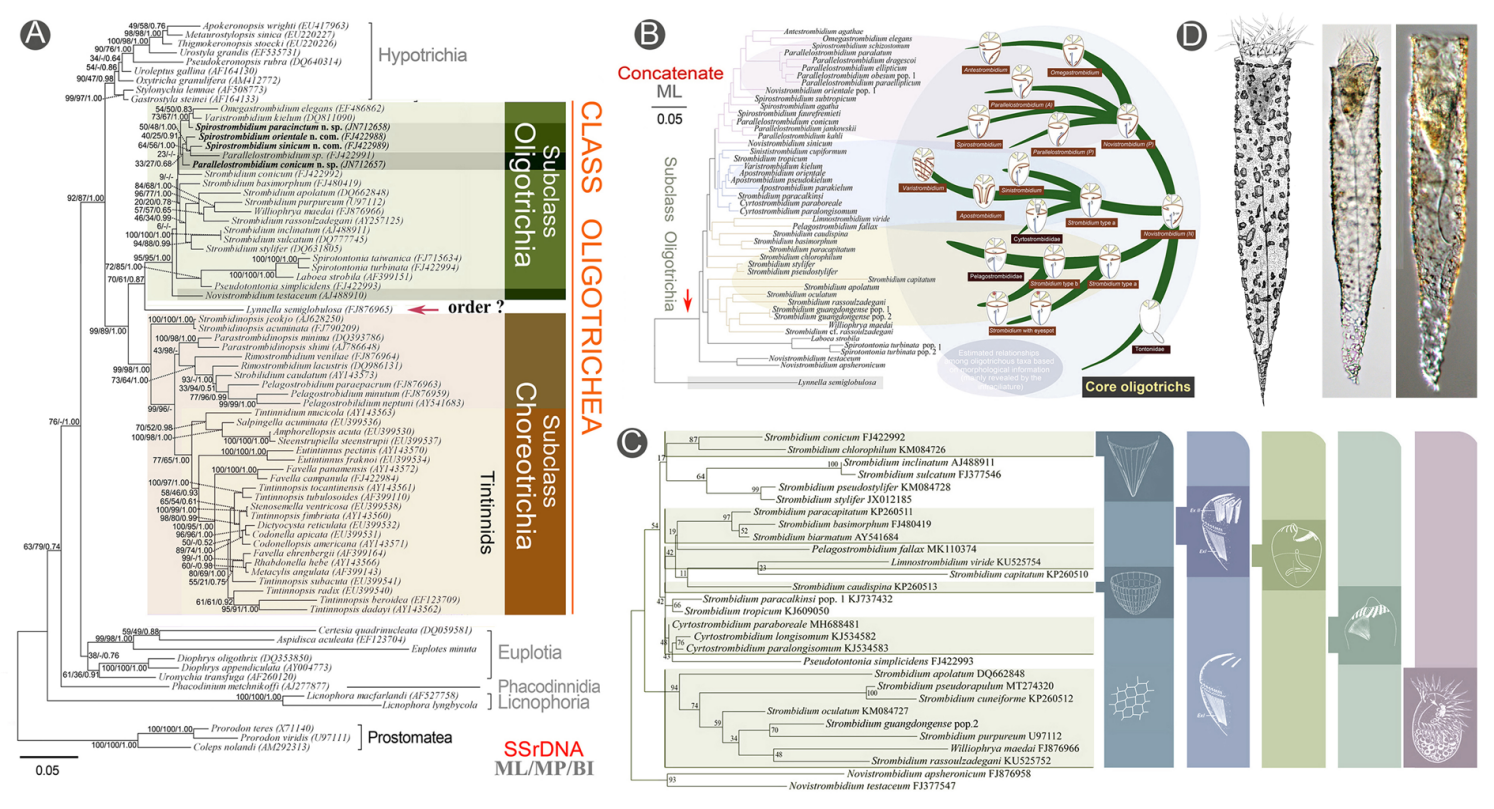
Phylogeny of the oligotrichs (*s.l.*). **A** Maximum-likelihood tree inferred from SSU rDNA sequences indicating the phylogenetic positions of species of the genera *Spirostrombidium* and *Parallelostrombidium* (Liu et al. 2013). **B** Hypothesized evolutionary trajectories of Oligotrichia based on morphological information (Song et al. 2023). **C** ML tree based on SSU rDNA sequences of species with *Strombidium*-type ciliary pattern (Song et al. 2023). **D** Morphology of *Tintinnopsis* cf. *radix* (Bai et al. 2020b)

Within Oligotrichia, 55 SSU rDNA, 58 ITS-5.8S rDNA, and 52 LSU rDNA have been sequenced and deposited in the GenBank database. Phylogenetic studies have revealed the following main findings: (1) The genera *Apostrombidium*, *Spirostrombidium*, and *Parallelostrombidium* are polyphyletic. In particular, *Parallelostrombidium* is divided into two distinct lineages. This finding is supported by the cell shape and somatic kinety pattern of its constituent species (Song et al. 2018b, 2019; Wang et al. 2018); (2) Morphological and phylogenetic analyses indicate that *Pelagostrombidium* and *Limnostrombidium* evolved within the family Strombidiidae, rather than evolving in parallel. This significance of the neoformation organelle has been overinterpreted as it does not warrant application as a family-level character (Song et al. 2019); (3) The spiraled girdle kinety observed in species of *Laboea* represents a synapomorphy rather than a convergent trait, and the tail structure was likely lost during the evolutionary history of this genus (Gao et al. 2009); (4) Four new genera have been established by the OUC-group: *Sinistrostrombidium*, *Antestrombidium*, *Williophrya*, and *Varistrombidium* (Liu et al. 2011b, 2015; Xu et al. 2011a).

Within the subclass Choreotrichia, 29 SSU rDNA sequences, 16 ITS-5.8S rDNA sequences, and 16 LSU rDNA sequences have been deposited in the GenBank database. The new findings include: (1) The ventral gap in *Parastrombidinopsis* and *Lynnella*, probably occurred secondarily during their evolution, representing a synapomorphic retrogression to the plesiomorphic (open) state (Song et al. 2018a); (2) The ciliary pattern of *Amphorellopsis acuta* is characterized by its evenly arranged somatic kineties; phylogenetic analysis suggests that it shares a very close common ancestor with aloricate choreotrichs. This could represent an evolutionary transitional group between the aloricate choreotrichs and tintinnids (Bai et al. 2020a); (3) The establishment of a new genus *Antetintinnopsis*, characterized by the presence of a unique long ciliary tuft (Wang et al. 2021c).

The phylogenetic relationships within oligotrichs are generally well-resolved. The primary task for systematists now is to discover novel forms. As oligotrichs represent a key marine planktonic group, further exploration of understudied oceanic zones (e.g., pelagic waters and polar seas) is expected to reveal new taxa awaiting description. These future findings will likely refine the current phylogenetic framework. Additionally, despite the large number of lorica-based morphotypes currently recognized, there is a lack of infraciliature and molecular information on tintinnids. Expanded sequence sampling, combined with a deeper understanding of their ultrastructure- and infraciliature-based taxonomy, is expected to significantly enhance our knowledge of the phylogeny of Oligotrichia and Choreotrichia in the future.

### Phylogenetic studies of the subclass Hypotrichia (Fig. 11)

The subclass Hypotrichia Stein, 1859, is considered to be one of the most complex and highly differentiated groups of ciliates. They exhibit high diversity with around 1,000 nominal species having been reported (Berger 1999, 2006, 2008, 2011; Foissner 2016). The systematics of hypotrichs has been the subject of extensive study, primarily due to their high morphological diversity, their wide range of morphogenetic patterns, and the incongruities between morphology/morphogenesis-based classifications and molecular phylogenies.

**Fig. 11:**
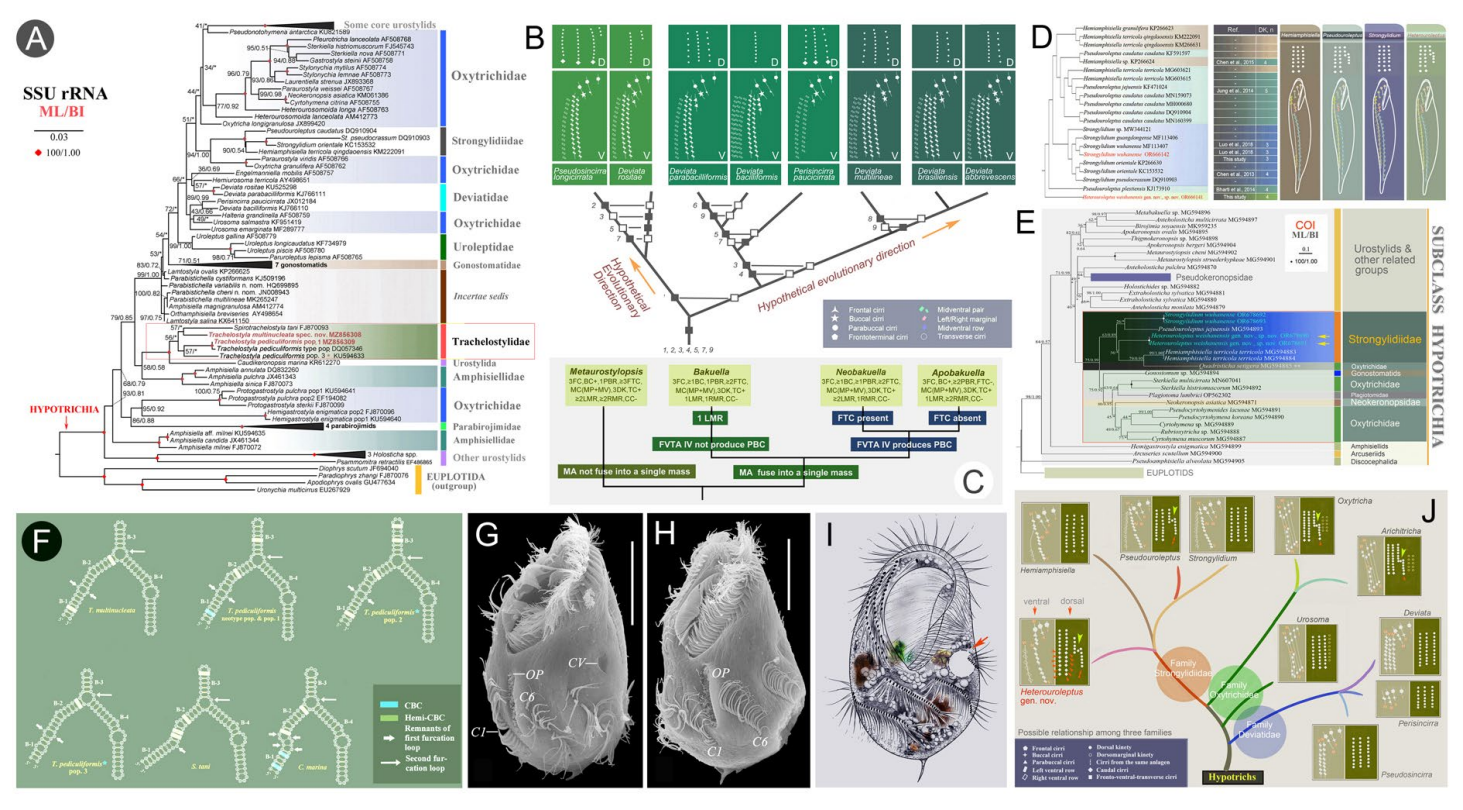
Phylogenetic studies of the subclass Hypotrichia. **A** Phylogenetic tree focusing on *Trachelostyla* inferred from SSU rDNA sequences (Zhang et al. 2022c). **B** Hypothetical systematic relationships within Deviatidae based on molecular and morphological data (Zhang et al. 2022b). **C** Comparison of some bakuellid genera (Zhang et al. 2020). **D** Morphogenetic information of four strongylidiid genera mapped onto SSU rDNA tree (Song et al. 2024). **E** Phylogenetic tree focusing on Strongylidiidae based on *COI* gene sequences (Song et al. 2024). **F** Secondary structure of helix B of five trachelostylids and *Caudikeronopsis marina* (Zhang et al. 2022c). **G–I** Morphogenesis (G, H) and morphology (I) of *Hypotrichidium tisiae* (Luo et al. 2022). **J** Morphogenetic information of strongylidiids based on the SSU rDNA tree (Song et al. 2024)

The OUC-group has analyzed the phylogenetic relationships of many representative hypotrich groups based on multi-gene sequence data alongside morphological and morphogenetic characters. One major group is the Urostylida, analyses of which supported the establishment of the new family Hemicycliostylidae and the exclusion of *Eschaneustyla* from the family Epiclintidae (Lyu et al. 2018). An integrative study of hypotrichs with a *Gonostomum*-patterned oral apparatus revealed that the endoral is more phylogenetically informative than the paroral, and that Schmidingerotrichidae is a distinct monophyletic family (Wang et al. 2021b). Analysis of the phylogenetically ambiguous family Deviatidae showed that its monophyly is well-supported and that deviatid members with dorsomarginal kineties are closely related (Zhang et al. 2022b).

A multifaceted approach to the taxonomically challenging genus *Trachelostyla* revealed that molecular data, including ITS2 secondary structures, are more suitable for delimiting *Trachelostyla* species than morphological characters (Zhang et al. 2022c). Similar investigations into Strongylidiidae supported its monophyly and indicated that the other two genera, *Hemiamphisiella* and *Pseudouroleptus*, should be subdivided due to their variable morphological characteristics (Song et al. 2024).

To date, the OUC-group has focused primarily on the phylogenetic assignments of species with ambiguous positions. For instance, the urostylid species *Holosticha viridis,* for which a detailed description had previously been lacking, was transferred to the newly established genus *Limnoholosticha* (Li et al. 2017). However, a more recent study revealed that this species actually represents a new genus and family, and may serve as an intermediate form between urostylids and dorsomarginalians (Song et al. 2021). An investigation of the atypical species *Clampia sinica*, which lacks the zigzag-patterned midventral cirri typical of urostylids, revealed that it represents a new species, genus, and family (Xu et al. 2022).

We also aim to resolve within-group relationships. One example is the family Spirofilidae, the monophyly of which has been questioned, thus providing the incentive to investigate its phylogeny. The systematics of *Hypotrichidium*, the type genus of Spirofilidae, has been debated for over a century. We carried out the first comprehensive ontogenetic study of *Hypotrichidium* enabling us to redefine its ciliary pattern. Combined with phylogenetic analyses, this supported the placement of Spirofilidae within Postoralida (Luo et al. 2022). The genus *Strongylidium*, assigned to Spirofilidae by Lynn (2008), has a long and complicated taxonomic history. We reactivated the family Strongylidiidae for *Strongylidium* and two closely related genera: *Pseudouroleptus* and *Hemiamphisiella* (Luo et al. 2018). A recent study showed that the spirofilid genera *Chaetospira* and *Stichotricha* clustered in a clade far from the Spirofilidae type genus, indicating that they should both be removed from Spirofilidae and supporting the validity of the family Chaetospiridae (Song et al. 2022).

Another noteworthy contribution is that, since 2017, we have submitted to the GenBank database over 230 marker gene sequences from about 130 populations spanning 21 families and 77 genera. Among these, three new families, 21 new genera, and 58 new species have been established. For data before 2017, please refer to Gao et al. (2016).

Despite Hypotrichia being one of the most extensively studied ciliate groups, numerous phylogenetic issues remain unresolved. For example, the internal evolutionary relationships of several species-rich families (e.g., Bakuellidae, Oxytrichidae, and Urostylidae), and genera (e.g., *Anteholosticha*, *Bakuella*, and *Oxytrichia*) are still ambiguous and have not been the subject of a comprehensive review (Jin et al. 2022; Lyu et al. 2018). In addition, the SSU rDNA is highly conserved in many groups, such as *Oxytricha*, necessitating further characterization of cryptic species or morphospecies (Fan et al. 2021). To resolve these issues and provide a more accurate classification, future integrative studies based on morphological, ontogenetic, and molecular data are urgently needed.

### Phylogenetic studies of the subclass Euplotia (Fig. 12)

The subclass Euplotia constitutes one of the most speciose groups of ciliates. For instance, the genus *Euplotes* alone comprises approximately 150 nominal species and has a cosmopolitan distribution (Curds 1975; Wicklow 1982). Members of Euplotia are characterized by a dorsoventrally flattened body shape, a well-developed adoral zone of membranelles, and significantly differentiated ventral cirri. According to the most recent classification systems, Euplotia contains two morphologically divergent orders: Discocephalida and Euplotida (Adl et al. 2012, 2019; Gao et al. 2016).

**Fig. 12:**
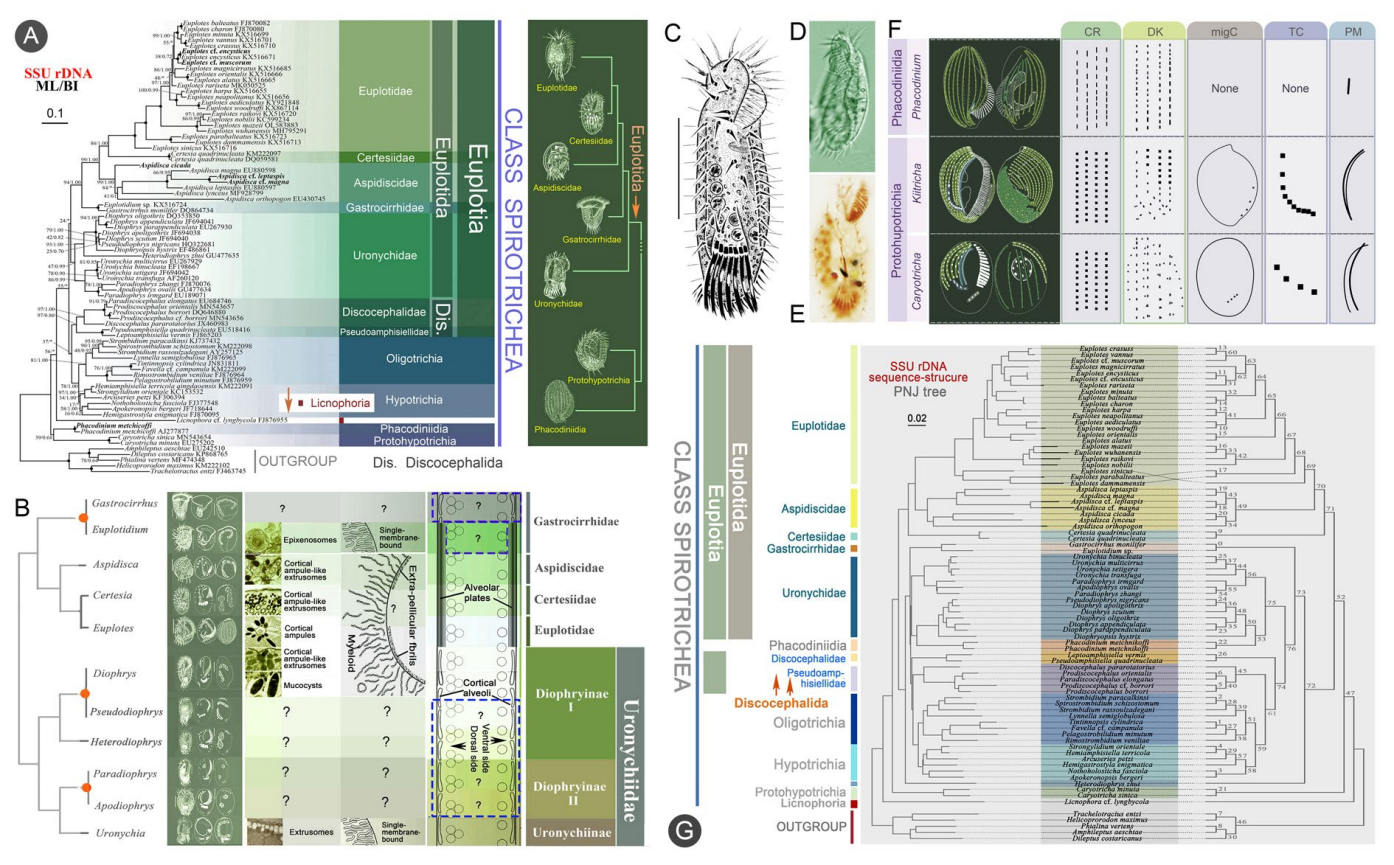
Phylogenetic studies of euplotians. **A** Maximum likelihood phylogenetic tree inferred from SSU rDNA sequences focusing on the subclass Euplotia (Lian et al. 2023). **B** Review of the published ultrastructural data and some speculative structures of Euplotida in the framework of SSU rRNA gene phylogeny, showing representative structures of genera and families (Dong et al. 2022). **C–E** Morphology of *Prodiscocephalus* cf. *borrori* in vivo and after protargol staining (Lian et al. 2020). **F** Morphological illustration of Euplotida (Lian et al. 2023). **G** Profile neighbor-joining (PNJ) phylogenetic tree inferred from SSU rDNA sequence–structure data of 80 taxa (Lian et al. 2023)

We have inferred the evolutionary relationships within Euplotia. Our findings suggest the following: (1) discocephalids should be placed outside of Euplotia, as a distinct order between Hypotrichia and Euplotia; and (2) Euplotidae and Aspidiscidae are the most recently diverged taxa within Euplotida, followed by Certesiidae and Gastrocirrhidae, whereas Uronychidae is the earliest-diverging branch (Lian et al. 2020, 2023; Miao et al. 2011; Shao et al. 2008; Yi et al 2009, 2012).

Phylogenetic analyses of euplotids reveal two significant evolutionary insights: (1) the *Diophrys* complex has a longer and more distinct evolutionary history than previously thought; and (2) the intraspecific divergence within *Aspidisca* is much greater than in closely related genera (Yi et al. 2009). Phylogenetic reconstruction of Euplotida using alpha-tubulin gene sequences reveals that this gene is a suitable phylogenetic marker for euplotids at the family level, that monophyletic paralogs in seven out of nine species indicate recent gene duplication events, and that Euplotidae exhibits more genetic diversity than is reflected in dargyrome patterns, habitat and SSU-rDNA phylogeny (Yi et al. 2012). Multigene phylogenetic analyses of the family Uronychiidae delineate four well-supported clades, which segregate into two subfamilies: Diophryinae and Uronychiinae (Huang et al. 2011). The ultrastructure of *Diophrys appendiculata* was subsequently documented and the systematics of the genus *Diophrys*, the subfamilies Diophryinae and Uronychiinae, and the order Euplotida were re-evaluated by integrating morphological, ultrastructural, and phylogenetic evidence (Dong et al. 2022). Furthermore, an integrative approach combining DNA data, ecological niche properties, and morphological characters was employed to reconstruct the evolutionary trajectories of the genus *Euplotes*. This comprehensive methodology also enabled an evaluation of the effectiveness of DNA taxonomy in determining species diversity within the genus (Zhao et al. 2018).

Recently, phylogenetic analyses of Euplotia were conducted using single-gene, concatenated four-gene, and SSU rDNA sequence-structure datasets. These findings allowed the probable evolutionary trajectories among families within Euplotida to be determined, as well as between euplotids and other spirotrich lineages, by integrating molecular, morphological, and morphogenetic data (Lian et al. 2023). Additionally, the OUC-group has deposited in the GenBank database over 250 maker gene sequences spanning seven families and 16 genera of euplotians. These include one new family (Pseudoamphisiellidae) and six new genera (*Heterodiophrys*, *Pseudodiophrys*, *Apodiophrys*, *Paradiscocephalus*, *Leptoamphisiella*, and *Pseudoamphisiella*) (Jiang et al. 2011; Jiang and Song 2010; Li et al. 2007, 2008a; Song 1996, 1997).

One of the most pressing issues in resolving the systematics of Euplotia concerns the phylogenetic relationship between Discocephalida and typical euplotids. Marker gene-based analyses position discocephalids at the base of the “core Euplotia” clade, suggesting that they represent either an outgroup or the ancestral lineage. However, this conclusion lacks strong morphological and morphogenetic support (Borror and Hill 1995; Yi et al. 2009). Furthermore, phylogenetic relationships among the six genera in the family Uronychiidae are difficult to infer using current datasets, highlighting the need for expanded multigene sequence sampling. In conclusion, while phylogenetic advancements over the past decades have substantiated the systematics of Euplotia at the family level, targeted investigations remain imperative to resolve persisting ambiguities in infra-familial classifications within this taxon, particularly through integrative approaches combining morphogenetic evidence with phylogenomic datasets.

### Phylogenomic studies of ciliates (Fig. 13)

Previous phylogenetic studies that relied mainly on a single locus or limited gene sets struggled to conclusively resolve evolutionary relationships, often yielding conflicting tree topologies (e.g. Lynn 2008; Gao et al. 2016). The advent of high-throughput sequencing has transformed phylogenomic approaches, facilitating comprehensive evolutionary reconstructions across various groups of organisms, including ciliates (Cai et al. 2022; Chen et al. 2018; Gentekaki et al. 2014).

**Fig. 13:**
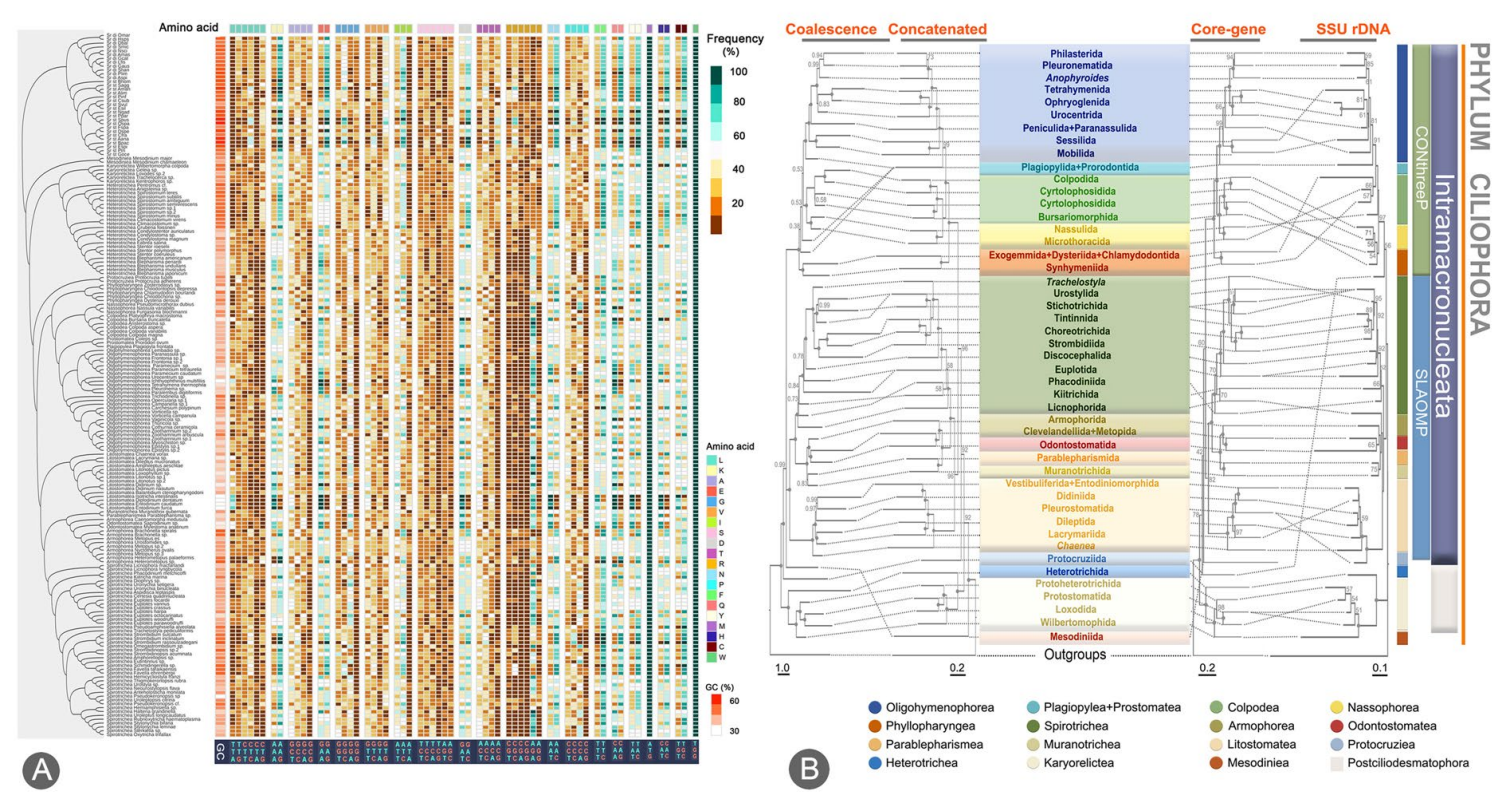
Codon usage bias and GC content of conserved gene families across different species (**A**) and a comparison of maximum likelihood (ML) phylogenies inferred from different datasets and algorithms (**B**). **A** Colored blocks at the top of the heatmap represent different amino acids, with synonymous codons grouped under the same color block. Purine and pyrimidine bases are distinguished by different colors. The tree on the left side corresponds to our phylogenomic results. Each tree is simplified to taxonomic orders and the same order in different trees is linked up by dashed lines. **B** Coalescent and concatenated phylogenies were inferred from the data matrix of 224 taxa (190 ciliates and 34 outgroup taxa) and 718 gene families, while the core-gene phylogeny was inferred from the top 200 gene families (Wang et al. In press)

Pioneering work by the OUC-group demonstrated that multi-omics data could be used to generate robust phylogenetic frameworks (Chen et al. 2015). Subsequent applications have achieved the following taxonomic advancements: (1) the reclassification of Synhymenia as a subclass within Phyllopharyngea rather than Nassophorea (Pan et al. 2019); (2) the taxonomic revision of *Protocruzia*, which removed it from Spirotrichea and elucidated its basal position within Intramacronucleata (Sheng et al. 2018; Zhang et al. 2024b); (3) the resolution of evolutionary relationships within Oligohymenophorea, confirming the monophyly of the subclass Peritrichia and establishing the new subclass Urocentria (Chen et al. 2020; Wang et al. 2021a; Zhang et al. 2023b); (4) the validation of the monophyly of Trichostomatia/Rhynchostomatia versus the polyphyly of Haptoria within Litostomatea (Zhang et al. 2024d; Zhou et al. 2024); (5) the proposal to subsume Odontostomatea, Muranotrichea and Parablepharismea into Armophorea (Zhang et al. 2024e); and (6) the reinterpretation of *Halteria grandinella* as an “oligotrich-like” hypotrich (Wang et al. 2019a). Additionally, some studies performed divergence time estimation and gene family evolution analyses based on reliable phylogenetic tree topologies derived from omics data (Jin et al. 2023, 2024; Li et al. 2021a; Zheng et al. 2022). However, existing studies are limited by insufficient taxon sampling and have failed to establish a comprehensive phylogenetic framework for the entire phylum Ciliophora.

To address this issue, we generated genomic and transcriptomic data from 52 ciliate species. By integrating several public datasets, we compiled the most extensive taxon sampling to date, comprising 190 species spanning 16 classes and 49 orders (Wang et al. submitted). Using 718 conserved gene families, we reconstructed the classification of ciliates, dividing Ciliophora into Mesodiniea and two major clades: Postciliodesmatophora and Intramacronucleata. The latter comprises Protocruziea plus two supergroups: CONthreeP (Colpodea, Oligohymenophorea, Nassophorea, Phyllopharyngea, Plagiopylea and Prostomatea) and SLAOMP (Spirotrichea, Litostomatea, Armophorea, Odontostomatea, Muranotrichea, and Parablepharismea). Our analysis strongly supports the recognition of Mesodiniea as a distinct class and Synhymenia as a distinct subclass, as well as the possible elevation of Armophorida to class level. We also compared the topologies of the molecular phylogenetic tree derived from two different approaches: supermatrix (concatenation) and supertree (coalescence). In addition, we identified a core set of 200 conserved gene families. All orthologs and multiple sequence alignments (MSAs) are publicly accessible for future phylogenomic research (http://106.13.47.147:8216/siglCellData).

Notably, certain clades (e.g., Mesodiniea) have unresolved placements due to limited data, which warrants further investigation. Furthermore, Lasek-Nesselquist and Johnson (2019) proposed that contamination by non-ciliate sequences would lead to *Mesodinium* being placed incorrectly in phylogenomic analyses. We echo these concerns and emphasize the need for rigorous contamination screening in future analyses.

### Challenges and future tasks

Decades of research into the systematics of the phylum Ciliophora, particularly the integration and refinement of morphological and molecular data, have largely clarified and stabilized the evolutionary relationships of higher-level taxa (i.e., classes and orders) within this group. The accuracy and reliability of this framework have been confirmed by numerous subsequent studies. However, challenges remain, particularly with regard to understudied taxa and those lacking sufficient molecular data. The main issues include the following:

1. The reliability of the species of many sequences registered in GenBank has not been verified. Over the past 30 years, there has been a rapid accumulation of gene sequences, particularly of the SSU rDNA sequences. However, many entries lack supporting morphological data and/or vouchered specimens, meaning that their identity cannot be verified. Furthermore, many sequences are mislabeled due to misidentified specimens, contributions from non-specialists, and unresolved cryptic diversity. These errors have resulted in significant phylogenetic artifacts, with conspecific sequences often appearing polyphyletic, artificially splitting naturally monophyletic groups.
2. There is a severe lack of molecular data for many taxa. As a classical research field, ciliate taxonomy has experienced a significant decline in research personnel over the past 50 years, resulting in a taxonomic impediment. At the same time, the application of new technologies and methods has revealed that the phylum Ciliophora still harbors a vast number of unknown and unresolved groups awaiting investigation, clarification, and discovery. Due to the shortage of researchers, there is a severe lack of understanding of many minor taxa (including numerous “lower groups”) and of endemic groups in specialized habitats. This has resulted in insufficient data being generated by the application of modern methods, including molecular sequences, and in the systematic placements of these groups being unreliable. Consequently, when reconstructing phylogenetic trees, the relationships between adjacent or related taxa often produce erroneous topologies.
3. The impact and challenges posed by omics technologies. As a prevailing trend, many phylogeneticists favor using “multi-gene” datasets. A clear underlying issue is that different genes with varying evolutionary rates are often assigned equal weighting when constructing phylogenetic trees, which is fundamentally problematic. As omics data becomes more widely available, a potential issue is that selecting inappropriate parts of genes (e.g., hypervariable or overly conserved regions) for inferring phylogenetic relationships could introduce new complications and confusion. In the absence of standardized guidelines and gene selection criteria, unchecked exploratory studies risk perpetuating hidden errors and methodological biases. This is a critical issue that requires heightened awareness among systematists.

The aforementioned issues should be of particular concern to researchers in this field. Specifically:

1. Critical use of GenBank data. Existing sequences should be employed with caution and scrutiny. Sequences of questionable reliability should either be excluded or clearly flagged as uncertain in phylogenetic trees, alerting readers to their potentially ambiguous status.
2. Expanding research on underexplored taxa. Intensive efforts are needed to study unknown and poorly known groups and rapidly generate essential systematic data, including molecular sequences.
3. Appropriate phylogenetic strategies. Tree-building methods and gene markers should be selected based on the specific taxonomic scope under investigation, rather than using a one-size-fits-all approach.

## Data Availability

Not applicable.
